# Multicentric Assessment of a Multimorbidity-Adjusted Disability Score to Stratify Depression-Related Risks Using Temporal Disease Maps: Instrument Validation Study

**DOI:** 10.2196/53162

**Published:** 2024-06-24

**Authors:** Rubèn González-Colom, Kangkana Mitra, Emili Vela, Andras Gezsi, Teemu Paajanen, Zsófia Gál, Gabor Hullam, Hannu Mäkinen, Tamas Nagy, Mikko Kuokkanen, Jordi Piera-Jiménez, Josep Roca, Peter Antal, Gabriella Juhasz, Isaac Cano

**Affiliations:** 1 Fundació de Recerca Clínic Barcelona - Institut d’Investigacions Biomèdiques August Pi i Sunyer Barcelona Spain; 2 Catalan Health Service Barcelona Spain; 3 Digitalization for the Sustainability of the Healthcare - Institut d'Investigació Biomèdica de Bellvitge Barcelona Spain; 4 Department of Measurement and Information Systems, Budapest University of Technology and Economics Budapest Hungary; 5 Department of Public Health and Welfare, Finnish Health and Welfare Institute Helsinki Finland; 6 Department of Pharmacodynamics, Faculty of Pharmacy, Semmelweis University Budapest Hungary; 7 NAP3.0-SE Neuropsychopharmacology Research Group, Hungarian Brain Research Program, Semmelweis University Budapest Hungary; 8 Department of Human Genetics and South Texas Diabetes and Obesity Institute, School of Medicine at University of Texas Rio Grande Valley Brownsville, TX United States; 9 Research Program for Clinical and Molecular Metabolism, Faculty of Medicine, University of Helsinki Helsinki Finland; 10 Faculty of Informatics, Telecommunications and Multimedia, Universitat Oberta de Catalunya Barcelona Spain; 11 Hospital Clínic de Barcelona Barcelona Spain; 12 Faculty of Medicine, Universitat de Barcelona Barcelona Spain

**Keywords:** health risk assessment, multimorbidity, disease trajectories, major depressive disorder

## Abstract

**Background:**

Comprehensive management of multimorbidity can significantly benefit from advanced health risk assessment tools that facilitate value-based interventions, allowing for the assessment and prediction of disease progression. Our study proposes a novel methodology, the Multimorbidity-Adjusted Disability Score (MADS), which integrates disease trajectory methodologies with advanced techniques for assessing interdependencies among concurrent diseases. This approach is designed to better assess the clinical burden of clusters of interrelated diseases and enhance our ability to anticipate disease progression, thereby potentially informing targeted preventive care interventions.

**Objective:**

This study aims to evaluate the effectiveness of the MADS in stratifying patients into clinically relevant risk groups based on their multimorbidity profiles, which accurately reflect their clinical complexity and the probabilities of developing new associated disease conditions.

**Methods:**

In a retrospective multicentric cohort study, we developed the MADS by analyzing disease trajectories and applying Bayesian statistics to determine disease-disease probabilities combined with well-established disability weights. We used major depressive disorder (MDD) as a primary case study for this evaluation. We stratified patients into different risk levels corresponding to different percentiles of MADS distribution. We statistically assessed the association of MADS risk strata with mortality, health care resource use, and disease progression across 1 million individuals from Spain, the United Kingdom, and Finland.

**Results:**

The results revealed significantly different distributions of the assessed outcomes across the MADS risk tiers, including mortality rates; primary care visits; specialized care outpatient consultations; visits in mental health specialized centers; emergency room visits; hospitalizations; pharmacological and nonpharmacological expenditures; and dispensation of antipsychotics, anxiolytics, sedatives, and antidepressants (*P*<.001 in all cases). Moreover, the results of the pairwise comparisons between adjacent risk tiers illustrate a substantial and gradual pattern of increased mortality rate, heightened health care use, increased health care expenditures, and a raised pharmacological burden as individuals progress from lower MADS risk tiers to higher-risk tiers. The analysis also revealed an augmented risk of multimorbidity progression within the high-risk groups, aligned with a higher incidence of new onsets of MDD-related diseases.

**Conclusions:**

The MADS seems to be a promising approach for predicting health risks associated with multimorbidity. It might complement current risk assessment state-of-the-art tools by providing valuable insights for tailored epidemiological impact analyses of clusters of interrelated diseases and by accurately assessing multimorbidity progression risks. This study paves the way for innovative digital developments to support advanced health risk assessment strategies. Further validation is required to generalize its use beyond the initial case study of MDD.

## Introduction

### Background

The co-occurrence of multiple chronic diseases, known as multimorbidity [1], affects 1 in 3 adults. Its prevalence rises with age, affecting 60% of individuals aged between 65 and 74 years and escalating to 80% among those aged ≥85 years [2]. Due to its association with poor prognosis, functional impairment, and reduced quality of life, multimorbidity is considered a global health care challenge [3,4] tied to complex clinical situations, leading to increased encounters with health care professionals, hospitalizations, and pharmacological prescriptions, resulting in a substantial rise in health care costs [5]. The emergence of multimorbidity is not arbitrary and frequently aligns with shared risk factors and underlying pathophysiological mechanisms [6-8] that result from complex interactions between genetic and environmental factors throughout the life span [9]. Perceiving diseases not in isolation but as integral components of a more extensive, interconnected system within the human body has led to the emergence of network medicine [10,11]. Network medicine analyzes disease co-occurrence patterns, aiming to understand the complex connections between diseases to uncover biomarkers, therapeutic targets, and potential interventions [12,13]. The studies investigating the temporal patterns of disease concurrence, or disease trajectories [14,15], rely on a pragmatic approach to this concept to yield a better understanding of the time-dependent relationships among diseases and establish a promising landscape to identify disease-disease causal relationships.

According to this paradigm, a disease-centered approach might lead to suboptimal treatment of patients with multiple chronic conditions, triggering the need to implement new tools to enhance the effectiveness of health services [16]. In this regard, multimorbidity-adjusted health risk assessment (HRA) tools [17-21], such as the morbidity groupers, are crucial for assessing the comprehensive health needs of patients with multimorbidity [22]. HRA uses algorithms and patient data to categorize individuals by risk, aiding health care professionals in customizing interventions, optimizing resource allocation, and enhancing patient outcomes through preventive care. HRA tools facilitate efficient case-finding and screening processes [23]. Case finding targets the individuals at high risk, which is crucial for specialized health care programs, whereas patient screening detects latent illnesses early, enabling cost-effective interventions to prevent disease progression and reduce health care demands.

However, despite their widespread use, prevailing population-based HRA tools such as the Adjusted Clinical Groups [24], Clinical Risk Groups [25], or Adjusted Morbidity Groups (AMG) [4,21] still do not incorporate information on disease trajectories in their calculations. The AMG system is currently used in Catalonia (Spain; 7 million inhabitants) for health policy and clinical purposes. Adding disease-disease association information to the AMG (or other morbidity groupers) may open new avenues for implementing epidemiological impact analyses concerning clusters of interrelated diseases. In addition, it may facilitate the construction of risk groups that accurately represent probabilities of developing new associated disease conditions [26] susceptible to early prevention.

### Objectives

While acknowledging current limitations, this study sought to explore the feasibility of incorporating procedures relevant to the study of disease trajectories [14,15] and novel techniques for analyzing dependency relationships between concomitant diseases [27,28] to improve the capabilities of the current morbidity groupers. This approach might better adjust the estimations of the burden of morbidity to clusters of diseases and improve the ability to anticipate the progression of multimorbidity.

We used major depressive disorder (MDD; F32-F33 in the *International Classification of Diseases, 10th Revision, Clinical Modification* [*ICD-10-CM*] [29]) as a use case due to its clinical relevance in multimorbidity management. However, this study pursued to showcase a methodology applicable beyond MDD, allowing for the assessment of the impact of multimorbidity across different clusters of diseases.

This paper describes the process of development and assessment of the Multimorbidity-Adjusted Disability Score (MADS) through an observational retrospective multicentric cohort study, showcasing a pioneering approach that integrates advanced techniques for analyzing disease associations, insights from the analysis of disease trajectories, and a comprehensive scoring method aimed at evaluating the disease burden. The MADS was designed to stratify patients with different health needs according to (1) the disease burden caused by MDD and its comorbidities on individuals and health systems and (2) the risk of morbidity progression and the onset of MDD comorbid conditions.

On the basis of the temporal disease maps among MDD and highly prevalent disease conditions [30] generated using Bayesian direct multimorbidity maps (BDMMs) [27,28], a promising method for filtering indirect disease associations in the context of the European Research Area on Personalized Medicine project “Temporal disease map based stratification of depression-related multimorbidities: towards quantitative investigations of patient trajectories and predictions of multi-target drug candidates” (TRAJECTOME) [31], we combined the probabilities of relevance (PRs) among MDD and its comorbid conditions with the disability weights (DWs) [32], documented in the 2019 revision of the Global Burden of Disease (GBD) study, to compute the MADS. We used the MADS to generate a risk pyramid and stratify the study population into 5 risk groups using different percentiles of MADS distribution. Finally, we analyzed the correspondence between the MADS risk groups and health outcomes through a cross-sectional analysis of mortality and use of health care resources and a longitudinal analysis of disease prevalence and incidence of new disease onsets. The clinical relevance of the identified risk groups was assessed through a multicentric assessment of the findings. To this end, MADS performance was analyzed using data from 3 independent European cohorts from the United Kingdom, Finland, and Spain including >1 million individuals.

## Methods

### Overview

**Figure 1:**
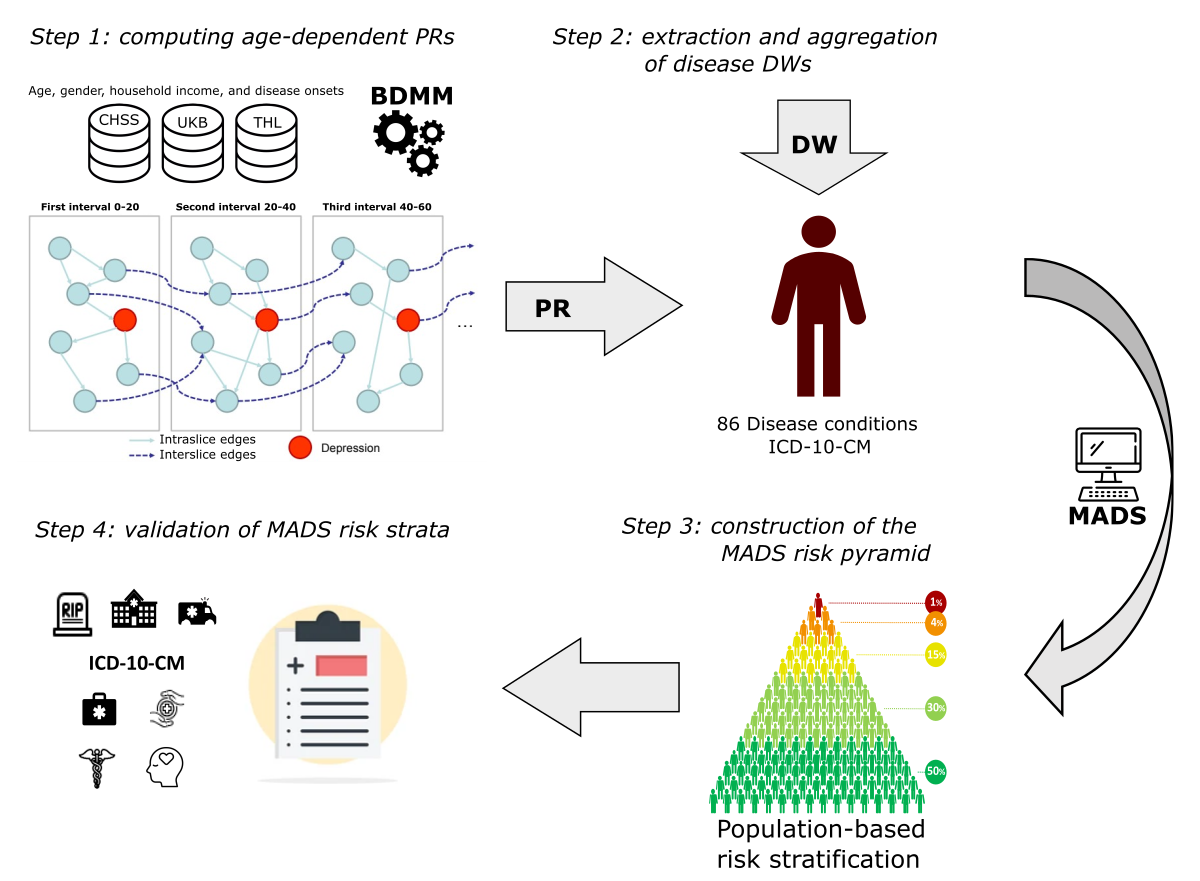
Workflow for building and assessing the Multimorbidity-Adjusted Disability Score (MADS). BDMM: Bayesian direct multimorbidity map; CHSS: Catalan Health Surveillance System; DW: disability weight; ICD-10-CM: International Classification of Diseases, 10th Revision, Clinical Modification; PR: probability of relevance; THL: Finnish Institute for Health and Welfare; UKB: UK Biobank.

The development and evaluation of the MADS involved the following steps (Figure 1).

Step 1 involved computing age-dependent disease-disease PRs using the BDMM method in 4 age intervals (0-20 years, 0-40 years, 0-60 years, and 0-70 years). This analysis resulted in an inhomogeneous dynamic Bayesian network that determined the PR for MDD against the most prevalent co-occurring diseases in the 3 European cohorts considered in TRAJECTOME, namely, the Catalan Health Surveillance System (CHSS) [33], the UK Biobank (UKB) [34], and the Finnish Institute for Health and Welfare (THL) [35] cohorts. The THL cohort amalgamates information from the FINRISK [36] 1992, 1997, 2002, 2007, and 2012; the FinHealth [37] 2017; and the Health [38] 2000 and 2011 studies.

In step 2, combining the PR of every disease condition assessed in the study with its corresponding DW extracted from the GBD 2019 study, we estimated the morbidity burden caused by MDD and its comorbid conditions. The MADS was computed following a multiplicative combination of the PR and DW of all the disease conditions present in an individual.

Step 3 involved using the MADS to stratify patients into different risk levels corresponding to different percentiles of the population-based risk pyramid of each patient cohort: (1) very low risk (percentile ≤50), (2) low risk (percentile 50 to percentile 80), (3) moderate risk (percentile 80 to percentile 90), (4) high risk (percentile 90 to percentile 95), and (5) very high risk (percentile >99).

Finally, in step 4, the correspondence between the MADS risk strata and health outcomes was analyzed through a cross-sectional analysis of use of health care resources, mortality, pharmacological burden, and health care expenditure and a longitudinal analysis of disease prevalence and incidence of new disease onsets. The results were validated through a multicentric replication of the findings in the 3 study cohorts including 1,041,014 individuals.

### Step 1: Computing Age-Dependent PRs

BDMMs were used to assess direct and indirect associations between MDD and 86 potential comorbid conditions. The set of 86 disease conditions considered in the study had a prevalence of >1% in all the study cohorts. The list of diseases and their associated *ICD-10-CM* [29] codes are shown in Multimedia Appendix 1.

This step considered information on *disease diagnosis* (disease conditions were cataloged using the first 3 characters of the *ICD-10-CM* codes), age at disease onset (the age at disease onset corresponds to the first diagnosis in a lifetime for each *ICD-10-CM* code), sex, and socioeconomic status (annual average total household income [before tax with copayment exemption] as a categorical variable with 3 categories: <€18,000 [US $19,565.30], €18,000-100,000 [US $19,565.30-108,696], and >€100,000 [US $108,696]).

BDMM analysis resulted in an inhomogeneous dynamic Bayesian network, which was used to compute temporal PR, ranging from 0 (no association) to 1 (strong association), for MDD in conjunction with sex, socioeconomic status, and the set of 86 predetermined diseases [30]. To construct the trajectories, the PR was calculated in 4 different age ranges: 0 to 20 years, 0 to 40 years, 0 to 60 years, and 0 to 70 years. The PRs calculated and used for MADS computation are reported in Multimedia Appendix 1. Further details regarding the core analysis conducted in TRAJECTOME can be found in the study by Juhasz et al [30].

### Step 2: Extracting and Aggregating Disease DWs

The MADS was developed by weighting the DWs of single diseases according to their estimated PRs against MDD. DWs indicate the degree of health loss based on several health outcomes and are used to indicate the total disability caused by a certain health condition or disease. Often, the DWs present specific disability scores tailored to the severity of the disease. The disease categories, severity distribution, and associated DWs used in this study were extracted from the GBD 2019 study and are reported in Multimedia Appendix 1.

DWs were extracted and aggregated as follows. First, we considered only the DWs of MDD and the set of 86 disease codes. Second, we considered the DWs of all the chronic conditions diagnosed in patients’ lifetime, whereas as the disability caused by acute illnesses is transitory, the DWs for the acute diseases diagnosed >12 months before the MADS assessment were arbitrarily set to 0 (no disability). Third, due to the unavailability of information on the severity of diagnoses, we determined the DWs of each disease condition by calculating the weighted mean of the DWs associated with the disease severity categories and their prevalence. In instances in which the severity distribution was not available, we computed the arithmetic mean of the DWs of each severity category. Fourth, we weighted the DWs according to the PR of each disease condition with respect to MDD. The PRs were adjusted according to the age of disease onset, discretized in the following intervals: 0 to 20 years, 20 to 40 years, 40 to 60 years, and >60 years.

As the DWs do not account for multimorbidity in their estimates, the use of DW independently can cause inaccuracies in the burden of disease estimations, particularly in aging populations that include large proportions of persons with ≥2 disabling disease conditions [39]. Consequently, we combined the DW and the PR for all the disease conditions present in 1 individual following a multiplicative approach (equation 1) [40], aggregating several DWs in a single score that accounts for the overall disability caused by numerous concurrent chronic conditions in which every comorbid disease increases the utility loss of a patient, although it is less than the sum of the utility loss of both diseases independently.

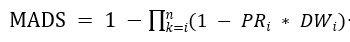



In equation 1, “DW” stands for disability weight, “PR” stands for probability of relevance, and “n” is the number of diseases present in 1 individual.

The MADS pseudocode is reported in Multimedia Appendix 1.

### Step 3: Constructing the MADS Risk Pyramid

Once calculated, the MADS was used to stratify patients in different levels of risk according to the percentiles of its distribution in the source population, producing the following risk pyramid: (1) very low risk (percentile ≤50), (2) low risk (percentile 50-percentile 80), (3) moderate risk (percentile 80-percentile 90), (4) high risk (percentile 90-percentile 95), and (5) very high risk (percentile >99).

### Step 4: Evaluating MADS Risk Strata

The clinical relevance of the risk strata was assessed through two interconnected analyses: (1) a cross-sectional analysis of health outcomes and (2) a longitudinal analysis of disease prevalence and incidence of new onsets.

#### Cross-Sectional Analysis of Health Outcomes and Use of Health Care Resources

To validate the results of the MADS, we conducted a cross-sectional analysis of clinical outcomes within the 12 months following the MADS assessment. The burden of MDD and its comorbidities on patients and health care providers, corresponding to each risk group of the MADS risk pyramid, was assessed using the following features (the parameters evaluated in each cohort may vary depending on the availability of the requested information in the source databases):

- Prescriptions for psycholeptic and psychoanaleptic drugs (information available in all the databases)—the prescribed medication was cataloged using the first 4 characters from Anatomical Therapeutic Chemical classification [41] codes, resulting in the following categories: *antipsychotics (N05A), anxiolytics (N05B), hypnotics and sedatives (N05C), and antidepressants (N06A)*
- Cost of the pharmacological prescriptions in euros (information available only in the CHSS and THL)
- Mortality rates (information available only in the CHSS and THL)
- Contacts and encounters with health care professionals (information available only in the CHSS), encompassing (1) primary care visits, (2) specialized care outpatient visits, (3) ambulatory visits in mental health centers, (4) emergency room visits, (5) planned and unplanned hospital admissions, and (6) admissions in mental health centers
- Total health care expenditure (information available only in the CHSS), including (1) direct health care delivery costs; (2) pharmacological costs; and (3) other billable health care costs such as nonurgent medical transportation, ambulatory rehabilitation, domiciliary oxygen therapy, and dialysis

We assessed the effect of sex and age, replicating the analyses disaggregated by sex and age. The age ranges were discretized into the following categories: 0 to 20 years, 20 to 40 years, 40 to 60 years, and >60 years.

#### Longitudinal Analysis of Disease Prevalence and Incidence of New Onsets

To address the age dependency of disease onsets, we performed a longitudinal analysis of the prevalence of a target disease and the incidence of new diagnostics within the 5 years following the MADS assessment.

We iteratively computed the MADS in 5-year intervals throughout the patients’ lives. Within each interval, the population was stratified based on the MADS distribution. Subsequently, within each risk tier, the prevalence of the target disease and the incidence of new disease onset over the subsequent 5 years were calculated. Only individuals with complete information for the next interval at each time point of the analysis were included.

In the analysis, we considered only the chronic disease conditions with a PR against MDD of ≥0.80 in at least 1 of the 4 age intervals assessed, namely, 0 to 20 years, 0 to 40 years, 0 to 60 years, and 0 to 70 years. It resulted in the following set of mental diseases—*MDD (F32-F33)*, *schizophrenia (F20)*, *bipolar disorder (F31)*, *anxiety-related disorders (F40-F41)*, *stress-related disorders (F43)*, and *mental disorders related to alcohol abuse (F10)*—and the following somatic diseases: *irritable bowel syndrome (K58)*, *overweight and obesity (E66)*, and *gastroesophageal reflux (K21)*.

### Data Sources

The study was conducted using data from 3 public health cohorts.

#### CHSS Cohort

The main cohort used in MADS development was extracted from the CHSS. Operated by a public single-payer system (CatSalut) [42] since 2011, the CHSS gathers information across health care tiers on the use of public health care resources, pharmacological prescriptions, and patients’ basic demographic data, including registries of 7.5 million citizens from the entire region of Catalonia (Spain). Nevertheless, for MADS development purposes, we considered only registry data from citizens residing in the entire Integrated Health District of Barcelona-Esquerra between January 1, 2011, and December 31, 2019 (n=654,913). To validate the results of the MADS, we retrieved additional information from the CHSS corresponding to the 12 months after the MADS assessment, from January 1, 2020, to December 31, 2020. It should be noted that all the deceased patients, in addition to those who moved their residence outside of the Integrated Health District of Barcelona-Esquerra between 2011 and 2019, were discarded from the MADS assessment analysis; the remaining subset of patients comprised 508,990 individuals.

#### UKB Cohort

The UKB data considered in this study contained medical and phenotypic data from participants aged between 37 and 93 years. Recruitment was based on National Health Service patient registers, and initial assessment visits were carried out between March 3, 2006, and October 1, 2010 (n=502,504). The analyzed data included disease diagnosis and onset time, medication prescriptions, and socioeconomic descriptors.

#### THL Cohort

The THL cohort integrates information from the FINRISK [36] 1992, 1997, 2002, 2007, and 2012; FinHealth [37] 2017; and Health [38] 2000/2011 studies. For the consensual clustering, 41,092 participants were used from Finnish population surveys. After data cleaning, 30,961 participants remained. These participants, aged 20 to 100 years, were chosen at random from the Finnish population and represented different parts of Finland.

Demographic information on the study cohorts is shown in the Results section.

### Ethical Considerations

As a multicentric study, TRAJECTOME accessed data from multiple cohorts, all subject to the legal regulations of their respective regions of origin, and obtained the necessary approvals from the corresponding ethics committees.

For the CHSS cohort, the Ethical Committee for Human Research at Hospital Clínic de Barcelona approved the core study of TRAJECTOME on March 24, 2021 (HCB/2020/1051), and subsequently approved the analysis for the generation and the assessment of the MADS on July 25, 2022 (HCB/2022/0720).

The UKB cohort received ethics approval from the National Research Ethics Service Committee North West–Haydock (reference 11/NW/0382).

The THL cohort integrates information from the FINRISK databases (1997 [ethical committee of the National Public Health Institute; statement 38/96; October 30, 1996], 2002 [Helsinki University Hospital, ethical committee of epidemiology and public health; statement 87/2001; reference 558/E3/2001; December 19, 2001], 2007 [Helsinki University Hospital, coordinating ethics committee; Dnro HUS 229/EO/2006; June 20, 2006], and 2012 [Helsinki University Hospital, coordinating ethics committee; Dnro HUS 162/13/03/11; December 1, 2011]), the FinHealth 2017 (Helsinki University Hospital, coordinating ethics committee; 37/13/03/00/2016; March 22, 2016), and the Health 2000 to 2011 databases (ethical committee of the National Public Health Institute, 8/99/12; Helsinki University Hospital, ethical committee of epidemiology and public health, 407/E3/2000; May 31, 2000, and June 17, 2011).

The ethics committees exempted the requirement to obtain informed consent for the analysis and publication of retrospectively acquired and fully anonymized data in the context of this noninterventional study.

All the data were handled in compliance with the General Data Protection Regulation 2016/679, which safeguards data protection and privacy for all individuals in the European Union (EU). The study was conducted in conformity with the Helsinki Declaration (Stronghold Version, Brazil, October 2013) and in accordance with the protocol and the relevant legal requirements (Law 14/2007 on Biomedical Research of July 3).

### Statistical Analysis

The results of the cross-sectional analysis of health outcomes and use of health care resources were evaluated through various metrics. Mortality rates were summarized as cases per 1000 inhabitants. In contrast, numeric health outcome variables were described by the average number of cases per person, per 100 inhabitants, or per 1000 inhabitants according to their prevalence. Average health care expenditures were reported in euro per person. Kruskal-Wallis tests, supplemented with Bonferroni-adjusted post hoc right-tailed Dunn tests, and pairwise Fisher exact tests were used to evaluate changes in the target outcomes across the risk pyramid tiers. Statistical significance was determined by considering a *P* value of <.05 in all analyses.

The results of the longitudinal analysis on disease prevalence and on the incidence of new disease onsets of MDD and 9 mental and somatic MDD-related chronic conditions (PR>0.80) were expressed in percentages and in per thousand (‰), respectively.

All the data analyses were conducted using R (version 4.1.1; R Foundation for Statistical Computing) [43]. The MADS algorithm was fully developed and tested in the CHSS database and transferred to the other sites using an R programming executable script.

The study is reported according to the STROBE (Strengthening the Reporting of Observational Studies in Epidemiology) [23] guidelines for observational studies.

## Results

### Sociodemographic Characteristics of the Study Cohorts

One of the first results was the characterization of the 3 study cohorts and comparison of the sociodemographic attributes of their MADS risk groups (Table 1). All the individuals were classified into distinct risk strata based on quantiles of MADS distribution within the source population, resulting in the formation of the subsequent risk pyramid: very-low-risk tier (percentile ≤50), low-risk tier (percentile 50 to percentile 80), moderate-risk tier (percentile 80 to percentile 90), high-risk tier (percentile 90 to percentile 95), and very high–risk tier (percentile >99).

**Table 1:** Demographic characteristics of each stratum of the Multimorbidity-Adjusted Disability Score (MADS) risk pyramid in the 3 study cohorts: Catalan Health Surveillance System (CHSS) [33], UK Biobank (UKB) [34], and Finnish Institute for Health and Welfare (THL) [35]^a^.

Risk pyramid tier and demographics				CHSS		THL		UKB
**All cases**								
	Participants, N			507,549		30,961		502,504
	Age (y), mean (SD)			45.36 (23.07)		64.27 (14.28)		61.48 (9.31)
	**Sex, n (%)**							
		Male	237,598 (46.8)		14,435 (46.61)		229,122 (45.6)	
		Female	269,951 (53.2)		16,526 (53.39)		273,382 (54.4)	
	**Household income, n (%)**							
		Low (<€18,000 [US $19,565.30])	262,753 (51.76)		11,489 (37.1)		117,737 (23.42)	
		Medium (€18,000-100,000 [US $19,565.30-$108,696])	223,369 (44)		10,025 (32.4)		358,492 (71.34)	
		High (>€100,000 [US $108,696])	21,427 (4.24)		9447 (30.5)		26,275 (5.24)	
	Major depressive disorder prevalence, n (%)			38,479 (7.58)		2287 (7.39)		53,466 (10.64)
**Very high risk (percentile >99)**								
	Participants, N			5651		310		5026
	Age (y), mean (SD)			55.74 (18.83)		68.83 (14.86)		61.7 (8.75)
	**Sex, n (%)**							
		Male	2322 (41.09)		129 (41.61)		2207 (43.89)	
		Female	3329 (58.91)		181 (58.39)		2819 (56.11)	
	**Household income, n (%)**							
		Low (<€18,000 [US $19,565.30])	4343 (76.86)		191 (61.61)		2285 (45.47)	
		Medium (€18,000-100,000 [US $19,565.30-$108,696])	1251 (22.13)		77 (24.84)		2620 (52.13)	
		High (>€100,000 [US $108,696])	57 (1.01)		42 (13.55)		121 (2.4)	
	Major depressive disorder prevalence, n (%)			3870 (68.48)		186 (60)		4370 (86.94)
**High risk (percentile 95 to percentile 99)**								
	Participants, N			22,894		1238		20,084
	Age (y), mean (SD)			60.08 (20.00)		65.12 (15.10)		63.2 (8.74)
	**Sex, n (%)**							
		Male	7170 (31.32)		559 (45.23)		7545 (37.57)	
		Female	15,724 (68.68)		679 (54.77)		12,539 (62.43)	
	**Household income, n (%)**							
		Low (<€18,000 [US $19,565.30])	14,568 (63.65)		690 (55.74)		7626 (37.97)	
		Medium (€18,000-100,000 [US $19,565.30-$108,696])	7946 (34.7)		327 (26.41)		12,003 (59.76)	
		High (>€100,000 [US $108,696])	380 (1.65)		221 (17.85)		455 (2.27)	
	Major depressive disorder prevalence, n (%)			18,368 (80.27)		734 (59.29)		19,039 (94.78)
**Moderate risk (percentile 80 to percentile 95)**								
	Participants, N			84,371		4644		75,378
	Age (y), mean (SD)			54.56 (21.87)		68.86 (14.77)		63.6 (9.02)
	**Sex, n (%)**							
		Male	34,462 (40.86)		2201 (47.4)		34,282 (45.48)	
		Female	49,909 (59.14)		2441 (52.6)		41,096 (54.52)	
	**Household income, n (%)**							
		Low (<€18,000 [US $19,565.30])	49,818 (59.05)		2285 (49.24)		23,208 (30.77)	
		Medium (€18,000-100,000 [US $19,565.30-$108,696])	32,822 (38.9)		1437 (30.93)		49,684 (65.93)	
		High (>€100,000 [US $108,696])	1731 (2.05)		920 (19.83)		2486 (3.3)	
	Major depressive disorder prevalence, n (%)			16,241 (19.25)		1367 (29.43)		25,776 (34.2)
**Low risk (percentile 50 to percentile 80)**								
	Participants, N			162,170		9,266		150,759
	Age (y), mean (SD)			47.66 (24.20)		66.16 (14.15)		62.2 (9.39)
	**Sex, n (%)**							
		Male	77,082 (47.53)		4132 (44.58)		70,550 (46.72)	
		Female	85,088 (52.47)		5137 (55.42)		80,209 (53.28)	
	**Household income, n (%)**							
		Low (<€18,000 [US $19,565.30])	85,936 (5300)		3623 (39.08)		36,773 (24.42)	
		Medium (€18,000-100,000 [US $19,565.30-$108,696])	71,429 (44.06)		3081 (33.25)		106,441 (70.59)	
		High (>€100,000 [US $108,696])	4805 (2.96)		2565 (27.67)		7545 (4.99)	
	Major depressive disorder prevalence, n (%)			0 (0)		0 (0)		2002 (1.3)
**Very low risk (percentile ≤50)**								
	Participants, N			232,463		15,503		251,257
	Age (y), mean (SD)			38.72 (20.72)		61.62 (13.55)		60.3 (9.22)
	**Sex, n (%)**							
		Male	116,562 (50.12)		7414 (47.83)		114,538 (45.59)	
		Female	115,901 (49.88)		8088 (52.17)		136,719 (54.41)	
	**Household income, n (%)**							
		Low (<€18,000 [US $19,565.30])	108,088 (46.48)		4700 (30.32)		47,845 (19.04)	
		Medium (€18,000-100,000 [US $19,565.30-$108,696])	109,921 (47.30)		5103 (32.92)		187,744 (74.72)	
		High (>€100,000 [US $108,696])	14,454 (6.22)		5699 (36.76)		15,668 (6.24)	
	Major depressive disorder prevalence, n (%)			0 (0)		0 (0)		2279 (0.9)

^a^The prevalence of depression was calculated considering both F32 and F33 International Classification of Diseases, 10th Revision, Clinical Modification diagnostic codes. Kruskal-Wallis tests were used to assess changes in the target outcomes according to the risk pyramid tiers (statistical significance: *P*<.05; H0=“all MADS risk groups have the same outcome distribution”; H1=“at least one MADS risk group has a different outcome distribution than the others”). *P*<.001 for age, sex, household income, and major depressive disorder prevalence for all cohorts.

It is imperative to underscore the fundamental distinctions in the cohorts under study to comprehend the inherent sociodemographic disparities across them. Specifically, the THL and UKB cohorts predominantly consist of data derived from biobanks, specifically focusing on the middle-aged and older adult population. In contrast, the CHSS cohort represents a population-based sample encompassing the entire population spectrum.

It is worth noting that a common pattern was observed among all the cohorts in the age distribution of the citizens at risk. Although the MADS is an additive morbidity grouper, it did not monotonically increase with age. Remarkably, a notable proportion of high-risk cases were observed within the age range of 40 to 60 years, when depression typically manifests for the first time on average.

A divergence in the sex distribution across the risk strata was observable and especially noticeable in the CHSS and UKB cohorts, where the morbidity burden associated with depression and its related diseases was amplified in women (*P*<.001). Similarly, the disability caused by depression and its comorbidities was larger in families with fewer economic resources (*P*<.001). Overall, the prevalence of MDD was greater in the UKB cohort than in the other cohorts. However, upon analyzing the allocation of the population with depression in the risk pyramid, a total of 57.79% (22,238/38,479) of individuals diagnosed with MDD were categorized in the “high”- and “very high” risk tiers in the CHSS cohort, whereas the proportion of individuals diagnosed with MDD who were allocated to the tip of the risk pyramid was 40.22% (920/2287) in the THL cohort and 43.78% (23,409/53,466) in the UKB cohort.

### Assessment of the MADS Risk Groups

#### Assessment of the PRs

**Figure 2:**
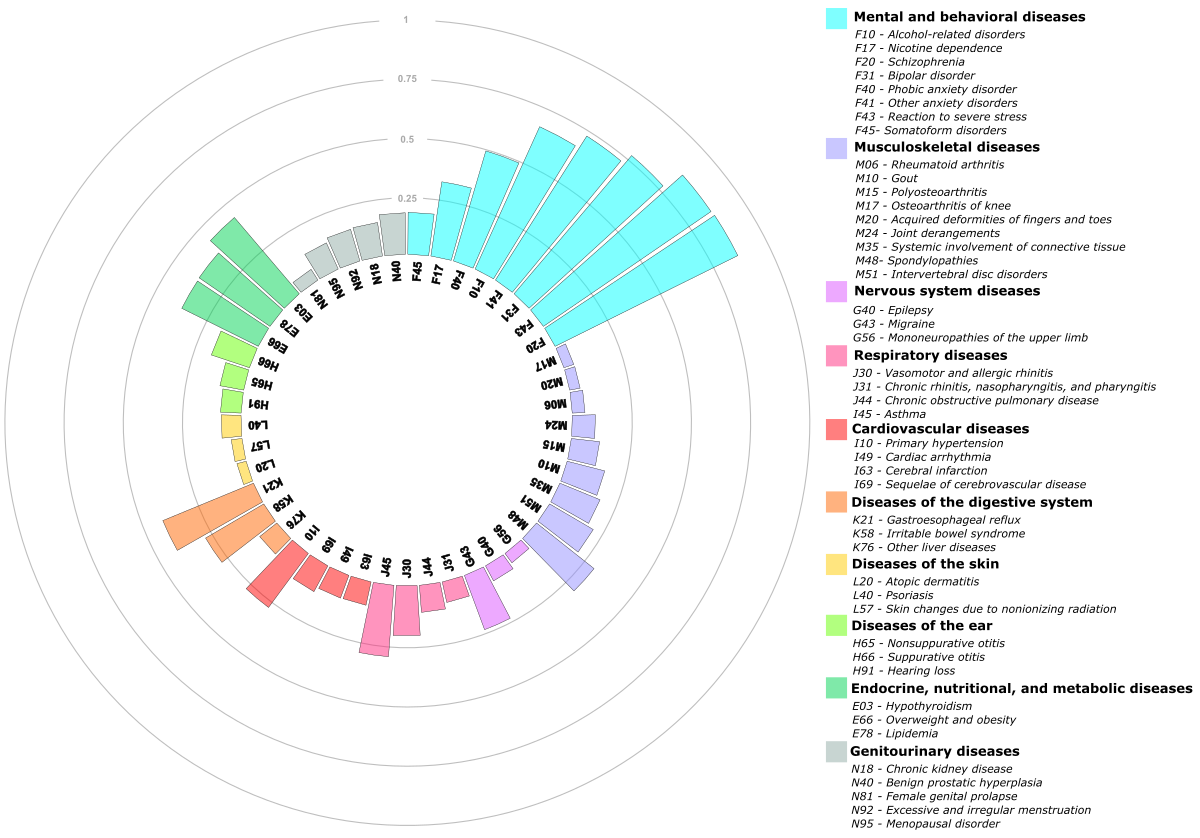
Average probabilities of relevance between major depressive disorder and 45 chronic conditions used to compute the Multimorbidity-Adjusted Disability Score.

Analyzing the relationship between MDD and the morbidities assessed in the study is essential to interpreting the MADS risk strata. This analysis revealed various relevant connections between MDD and the diseases investigated, encompassing both acute and chronic conditions, with the latter being particularly noteworthy due to their nontransient nature. Notably, the cluster of mental and behavioral disorders showed the highest average PRs in depression. However, relevant associations also emerged among MDD and specific chronic somatic diseases affecting multiple organic systems (Figure 2).

#### Use of Health Care Resources

The impact of MADS risk groups on health care systems was evaluated by investigating the correlation between the MADS risk categories and the use of health resources over the 12-month period following the MADS assessment within the CHSS cohort (Table 2). The results revealed significantly different distributions of the assessed outcomes across the MADS risk tiers, including primary care visits (*P*<.001), specialized outpatient visits (*P*<.001), emergency room visits (*P*<.001), hospital admissions (*P*<.001), and ambulatory visits in mental health centers (*P*<.001), as well as the pharmacological burden (*P*<.001). Furthermore, the results of the pairwise comparisons between adjacent risk tiers illustrated a substantial and gradual pattern of increased health care use as individuals progress from lower MADS risk tiers to higher MADS risk tiers, reflecting an escalation in health care needs and requirements. Overall, patients with higher MADS scores exhibited a greater likelihood of experiencing morbidity-related adverse events, which subsequently leads to recurrent interactions with health care systems across multiple levels.

**Table 2:** Use of health care resources over 12 months in each stratum of the Multimorbidity-Adjusted Disability Score risk pyramid for the Catalan Health Surveillance System cohort^a^.

Risk pyramid tier	Primary care visits (visits per person)	Specialized outpatient visits (visits per person)	Emergency room visits (visits per 100 inhabitants)	Hospital admissions (admissions per 100 inhabitants)	Mental health visits (visits per 100 inhabitants)	Number of prescriptions (prescriptions per person)
Very high risk (percentile >99)	12.50	3.07^b^	135.00^b^	28.50^b^	554.00^b^	8.02^b^
High risk (percentile 95 to percentile 99)	11.90^b^	2.56^b^	87.20^b^	20.60^b^	136.00^b^	7.48^b^
Moderate risk (percentile 80 to percentile 95)	9.03^b^	1.82^b^	61.90^b^	14.50^b^	44.20^b^	5.11^b^
Low risk (percentile 50 to tpercentile 80)	6.21^b^	1.21^b^	42.40^b^	8.87^b^	15.10^b^	3.20^b^
Very low risk (percentile ≤50)	2.96	0.50	23.40	3.25	5.96	1.07

^a^Kruskal-Wallis tests were used to assess changes in the target outcomes according to the risk pyramid tiers (*P* value). Subsequent pairwise comparisons between each risk tier and the next level of less risk were conducted using right-tailed Dunn post hoc tests (statistical significance: *P*<.05).

^b^*P*<.001.

#### Mortality and Health Care Expenditure

We conducted a cross-sectional analysis investigating mortality rates and the health care expenditure within the 12 months following the MADS assessment, expressed as the average health care expenditure per capita and differentiating between pharmaceutical and nonpharmaceutical costs within the CHSS and THL cohorts (Table 3). Significant variations in mortality rates were observed across the risk pyramid tiers (*P*<.001), with rates in the high-risk strata being markedly elevated (ranging from 5 to 20 times depending on the cohort) compared to those for low-risk individuals. Furthermore, the distribution of average health care expenditures per person was significantly different among the risk tiers, with both pharmacological and nonpharmacological expenses demonstrating disparities (*P*<.001). Pairwise comparisons further indicated that individuals at the highest-risk tier incurred substantially greater health care costs than those at the lowest tier, reflecting a gradient of financial impact correlated with increased risk levels.

**Table 3:** Mortality rates and pharmacological and nonpharmacological health care expenditure in euro over 12 months in each stratum of the Multimorbidity-Adjusted Disability Score risk pyramid in the Catalan Health Surveillance System (CHSS) [33] and Finnish Institute for Health and Welfare (THL) [35]^a^.

Risk pyramid tier	Mortality (cases per 1000 inhabitants)			Pharmacological expenditure in euro per person, mean (SD)			Hospitalization expenditure in euro per person, mean (SD)			Total expenditure in euro per person—CHSS, mean (SD)
	CHSS	THL	CHSS		THL	CHSS		THL		
Very high risk (percentile >99)	46.2^b^	36.0^b^	1214^b^		966	539^b^		270	12,517^b^	
High risk (percentile 95 to percentile 99)	41.5^b^	33.7^b^	772^b^		1131^b^	383^b^		340^b^	8404^b^	
Moderate risk (percentile 80 to percentile 95)	25.5^b^	32.2^b^	485^b^		1077^b^	270^b^		254^b^	5209^b^	
Low risk (percentile 50 to percentile 80)	11.5^b^	14.8^b^	292^b^		810^b^	165^b^		185^b^	3075^b^	
Very low risk (percentile ≤50)	2.57	7.3	99		363	60		123	1192	

^a^Kruskal-Wallis tests were used to assess changes in the target outcomes according to the risk pyramid tiers (*P* value). Subsequent pairwise comparisons between each risk tier and the next level of less risk were conducted using right-tailed Dunn post hoc tests. Pairwise comparisons of Fisher exact tests were used to assess changes in mortality rates. Statistical significance: *P*<.05.

^b^*P*<.001.

#### Pharmacological Burden

This study also examined the pharmacological burden on individuals after 12 months following the MADS assessment (Table 4). The data analysis revealed distinct patterns of medication use across the risk tiers, with significant differences in the use of antidepressants, antipsychotics, anxiolytics, and sedatives (*P*<.001 in all cases). This trend, consistently observed across the 3 cohorts, was further emphasized by pairwise comparisons between adjacent risk levels, which revealed a strong positive correlation between higher-risk strata and increased pharmaceutical consumption. This upward trend in medication use forms a clear gradient, demonstrating that individuals in progressively higher-risk tiers face substantially greater pharmaceutical needs.

**Table 4:** Prescription of depression-related pharmacological treatments over 12 months in each stratum of the Multimorbidity-Adjusted Disability Score risk pyramid in the Catalan Health Surveillance System (CHSS) [33], UK Biobank (UKB) [34], and Finnish Institute for Health and Welfare (THL) [35]^a^.

Risk pyramid tier	Antipsychotics (N05A; prescriptions per person)				Anxiolytics (N05B; prescriptions per person)				Hypnotics and sedatives (N05C; prescriptions per person)				Antidepressants (N06A; prescriptions per person)		
	CHSS	THL	UKB	CHSS		THL	UKB	CHSS		THL	UKB	CHSS		THL	UKB
Very high risk (percentile >99)	0.75^b^	0.60^b^	0.33^b^	0.47		0.21^b^	0.27^b^	0.15^b^		0.14^b^	0.24^b^	0.79^b^		0.43^b^	0.80^b^
High risk (percentile 95 to percentile 99)	0.20^b^	0.27^b^	0.18^b^	0.46^b^		0.19^b^	0.20^b^	0.10^b^		0.12^b^	0.19^b^	0.66^b^		0.41^b^	0.71^b^
Moderate risk (percentile 80 to percentile 95)	0.07^b^	0.08^b^	0.15^b^	0.28^b^		0.08^b^	0.16^b^	0.05^b^		0.10^b^	0.18^b^	0.27^b^		0.27^b^	0.54^b^
Low risk (percentile 50 to percentile 80)	0.03^b^	0.03^b^	0.13^b^	0.14^b^		0.04^b^	0.12^b^	0.02^b^		0.07^b^	0.13^b^	0.08^b^		0.11^b^	0.36^b^
Very low risk (percentile ≤50)	0.01	0.01	0.11	0.04		0.02	0.09	0.01		0.04	0.10	0.02		0.06	0.26

^a^For recurrently dispensed medication, only the first prescription was considered in the analysis. Kruskal-Wallis tests were used to assess changes in the target outcomes according to the risk pyramid tiers (*P* value). Subsequent pairwise comparisons between each risk tier and the next level of less risk were conducted using right-tailed Dunn post hoc tests. Statistical significance: *P*<.05.

^b^*P*<.001.

To evaluate the influence of age and sex on the outcomes examined in this section, we replicated all the previously presented results categorizing the outcomes by sex and age and reported them in Multimedia Appendix 1. The results suggest that the morbidity burden in individuals might be a primary driver influencing the occurrence of adverse health events and the heightened use of health care resources.

#### Multimorbidity Progression

We analyzed the prevalence and incidence of new MDD-associated diagnoses and the relevant comorbid conditions in 5-year intervals after the MADS assessment for depression throughout the patients’ life span (Multimedia Appendix 2), allowing for a comprehensive examination of multimorbidity progression over time.

Multimedia Appendix 2 shows the current disease prevalences expressed in percentages and the incidence of new disease onsets across an interval of 5 years after the MADS assessment expressed in per thousand. Multimedia Appendix 2 also showcases the results for MDD and 9 mental and somatic MDD-related (PR>0.80) chronic conditions assessed independently in the 3 study cohorts, namely, CHSS, THL, and UKB, and in 4 time points, that is, ages of 20 years, 40 years, 60 years, and 70 years, corresponding to the intervals in which the PRs were recalculated. A continuous assessment of these outcomes is reported in Multimedia Appendix 1.

In general, both MDD and the comorbid conditions investigated in this study exhibited a positive correlation between the MADS risk tiers and the current prevalence and incidence of new disease onsets within a subsequent 5-year interval. This is evident from the table. Notably, the highest disease prevalence and incidence values consistently appeared in the high- and very-high-risk tiers. In addition, there was a discernible pattern of well-stratified values across these risk tiers within the same age ranges, underlining significantly elevated prevalence rates of the studied diseases compared to the population average within the high-risk groups. Age also emerged as a pivotal determinant influencing disease onset, delineating unique patterns across various disorders. Notably, conditions such as gastroesophageal reflux and overweight consistently exhibited ascending trends in both incidence and prevalence throughout individuals’ life spans. Conversely, severe afflictions such as schizophrenia, bipolar disorder, and alcohol abuse reached their zenith in prevalence and incidence during middle-aged adulthood followed by a decline, possibly indicating an association with premature mortality. Moreover, anxiety- and stress-related disorders showed their highest incidence rates during youth and early adulthood.

The consistency of the findings illustrated in Multimedia Appendix 2 remained robust across all 3 study cohorts despite their significant demographic differences, described in Table 1. These heterogeneities resulted in disease prevalence discrepancies among cohorts, as vividly portrayed in Multimedia Appendix 2. Among the most relevant cases, there was an elevated prevalence of schizophrenia in the THL cohort in comparison with the CHSS and UKB cohorts. In this particular case, patients with schizophrenia constituted 100% of the very high–risk group in adulthood. Such differences in disease prevalence among cohorts may influence distinct health outcomes, particularly for the citizens allocated to the apex of the Finnish risk pyramid, as observed in the pharmacological and hospitalization expenditure outcomes reported in Table 3.

## Discussion

### Principal Findings

The MADS seems to provide a novel and more comprehensive understanding of the complex nature of depression-related multimorbidity. This approach recognizes that individuals with depression often experience a range of comorbid conditions that may manifest and evolve differently over time. By capturing this dynamic aspect, the MADS offers a nuanced assessment beyond a mere checklist of discrete disorders. The novelty of the MADS approach lies in its capability to serve as the first morbidity grouper that incorporates information on disease trajectories while improving the filtering of indirect disease associations using BDMMs.

In addition to capturing disease-disease associations, the MADS endeavors to gauge their impact within the system by leveraging well-established DWs. However, despite achieving success in fulfilling the study’s objectives, it is crucial to acknowledge that this approach carries inherent limitations, as will be elaborated on in the subsequent sections of this discussion.

In this investigation, we unearthed robust correlations between the MADS risk strata and the extent of deleterious impact caused by MDD and its comorbid conditions. Such associations indicate the presence of specific health risks and an escalated use of health care resources. Furthermore, a positive association emerged between the levels of pharmacological and nonpharmacological health care expenditures and the different tiers of MADS risk. In addition, the analysis revealed an augmented risk of disease progression within the high-risk groups (high and very high risk), as indicated by a heightened incidence of new-onset depression-related illnesses within a 12-month period after the MADS assessment. Similarly, mortality rates exhibited elevated values in these high-risk groups.

The findings presented in this study are underpinned by the complementary studies conducted within the TRAJECTOME project [30] that have established a better understanding of the complex multimorbidity landscape associated with MDD across an individual’s life span, encompassing modifiable and genetic risk factors.

### Limitations of This Approach

Despite meeting expectations and validating the hypothesis through which the study was conceived, the authors acknowledge a series of limitations leading to suboptimal results and limited potential for adaptation and generalization that should be undertaken to bring the MADS, or an indicator derived from it, to short-term real-world implementation.

In this research, the use of estimations of mean DW [44] to assess the burden of disease conditions achieved desirable results and was conceptually justified, but it undoubtedly exhibited significant limitations. In an ideal clinical scenario, each disease diagnosis indicated in the patient’s electronic medical record should be characterized by three key dimensions: (1) severity of the diagnosis, (2) rate of disease progression, and (3) impact on disability. However, the degree of maturity for characterizing the last 2 dimensions—disease progression and disability—is rather poor because of the complexities involved in their assessment. In other words, the authors acknowledge the weakness associated with the current use of DW. However, they stress the importance of incorporating such dimensions in future evolutions of the MADS.

A noteworthy aspect that should be acknowledged is that factors such as the advancements in diagnostic techniques, the digitization of medical records, and the modifications in disease taxonomy and classification over time have contributed to a more exhaustive documentation of the disease states in the most recent health records. Consequently, this fact could lead to imprecisions in estimating the disease onset ages in older individuals.

### Insights and Potential Impact of the MADS in Multimorbidity Management

The results reported in this study not only reaffirm the well-established link between multimorbidity and adverse outcomes, such as a decline in functional status, compromised quality of life, and increased mortality rates [45], but also shed light on the significant burden imposed on individuals and health care systems. From the population-based HRA perspective, the strain on resource allocation and overall health care spending is a pressing concern that necessitates effective strategies for addressing and managing multimorbidity [46]. In this context, assessing individual health risks and patient stratification emerge as crucial approaches that enable the implementation of predictive and preventive measures in health care.

While population-based HRA tools such as Adjusted Clinical Groups, Clinical Risk Groups, or AMG have traditionally addressed this aspect, the MADS is designed to complement rather than replace those tools. This study aimed to test a method to refine existing HRA tools by aligning them with the principles of network medicine, thereby merging traditional HRA with the practical application of network medicine insights. This innovative approach holds the promise of unlocking new potential advantages and capabilities.

The strength of the MADS approach lies in using disease-disease associations drawn from the analysis of temporal occurrence patterns among concurrent diseases. This virtue allows the MADS to refine the analysis of the morbidity burden by focusing on clusters of correlated diseases, which in turn can aid in developing more tailored epidemiological risk-related studies. This refined analysis might also assist in resource allocation and inform health care policies for targeted patient groups with specific needs. Moreover, this approach holds promise for potential extrapolation to other noncommunicable disease clusters such as diabetes, cardiovascular ailments, respiratory diseases, or cancer. By leveraging this targeted approach, the MADS can be adapted to other disease clusters with shared characteristics, enabling a more precise assessment of disease burden and comorbidity patterns and thereby generating multiple disease-specific indexes.

Notably, when considering information derived from disease co-occurrence patterns, the presence or absence of certain diseases seems to correlate with the risk of developing related comorbid conditions, as elucidated in Multimedia Appendix 2. This highlights the potential for a nuanced understanding of disease relationships and their impacts on health outcomes and to implement preventive interventions to mitigate their effect. Moreover, the findings of this study highlight the potential of preventive strategies targeted at mental disorders, including substance abuse disorders, depressive disorders, and schizophrenia, to reduce the incidence of negative clinical outcomes in somatic health conditions. These important implications for clinical practice call for a comprehensive and interdisciplinary approach that bridges the gap between psychiatric and somatic medicine. By developing cross-specialty preventive strategies, health care professionals can provide more holistic and effective care for individuals with complex health needs, ensuring that their mental and physical health are adequately addressed [47].

This study provided good prospects of using disease trajectories to enhance the performance of existing state-of-the-art morbidity groupers such as AMG. Recognized for its transferability across EU regions by the EU Joint Action on implementation of digitally enabled integrated person-centered care [48], AMG stands out due to its stratification capabilities, adaptability, and distribution as open-source software, providing several advantages over its commercial counterparts. The AMG system uses disease-specific weighting derived from statistical analysis incorporating mortality and health care service use data. This method addresses the primary drawback identified in the MADS approach inherent to the use of DW while enabling the development of adaptable tools that align with the unique characteristics of each health care system. Consequently, it allows for the adjustment to the impact of specific disease conditions within distinct regions and enhances the overall applicability and adaptability of the tool. In this regard, this study offered promising insights aligned with the developers’ envisioned future features for integration into the AMG system. Serving as a proof of concept, it highlighted the potential improvements achievable within AMG by leveraging disease-disease associations, thereby shaping the road map for further AMG development.

### MADS Integration in Precision Medicine: Advancing Toward Patient-Centric Strategies

By assessing whether the MADS is appropriate for the stratification of depression-related multimorbidity, we attempted to confirm its potential for contributing to precision medicine [49]. In the clinical arena, identifying individuals at elevated risk and customizing interventions enable health care providers to intervene proactively, potentially preventing or lessening disease progression and enhancing patient outcomes. These strategies not only yield immediate value in terms of improved patient care but also lay the foundation for the broader adoption of integrated care and precision medicine, particularly in the management of chronic conditions [50].

Incorporating systems medicine [51] methodologies and ITs has prompted significant shifts in clinical research and practice, paving the way for holistic approaches, computational modeling, and predictive tools in clinical medicine. These advancements are driving the adoption of clinical decision support systems, which use patient-specific data to generate assessments or recommendations, aiding clinicians in making informed decisions. It is well established that, to improve predictive precision and aid clinical decision-making, implementing comprehensive methodologies that consider various influencing factors from multiple sources in patient health could enhance individual prognosis estimations [52].

This integration might facilitate predictive modeling methodologies for personalized risk prediction and intervention planning. This approach, known as multisource clinical predictive modeling [53,54], enables the integration of (1) health care data and health determinants from other domains, including (2) population health registry data; (3) informal care data (including patients’ self-tracking data, lifestyles, environmental and behavioral aspects, and sensors); and, ideally, (4) biomedical research omics data. In this paradigm, it is crucial to acknowledge the pivotal role that multimorbidity groupers play in capturing the clinical complexity of individuals. Previous research [53,54] has highlighted the synergy between patient clinical complexity (eg, AMG) and acute episode severity, correlating with higher risks of adverse health events. This opens avenues for further research, exploring how adjusted morbidity indicators such as the MADS can significantly contribute to predictive modeling, aiming at supporting the implementation of cost-effective, patient-centered preventive measures to manage patients with chronic diseases and potentially delay or prevent their progression to the highest-risk levels in the stratification pyramid [55].

### Conclusions

The MADS showed to be a promising approach to estimate multimorbidity-adjusted risk of disease progression and measure MDD’s impact on individuals and health care systems, which could be tested in other diseases. The novelty of the MADS approach lies in its unique capability to incorporate disease trajectories, providing a comprehensive understanding of depression-related morbidity burden. In this regard, the BDMM method played a crucial role in isolating and identifying true direct disease associations. The results of this study pave the way for the development of innovative digital tools to support advanced HRA strategies. Nevertheless, clinical validation is imperative before considering the widespread adoption of the MADS.

## Appendix

Multimedia Appendix 1 Supplementary material encompassing the tables and figures from the cross-sectional and longitudinal analyses of outcomes, along with the disability weights and probabilities of relevance, as well as the Multimorbidity-Adjusted Disability Score (MADS) pseudocodes.

Multimedia Appendix 2 Longitudinal analysis of disease prevalence and incidence of new disease onsets in the Catalan Health Surveillance System, UK Biobank, and Finnish Institute for Health and Welfare.

## Abbreviations

AMG: Adjusted Morbidity Groups
BDMM: Bayesian direct multimorbidity map
CHSS: Catalan Health Surveillance System
DW: disability weight
EU: European Union
GBD: Global Burden of Disease
ICD-10-CM: International Classification of Diseases, 10th Revision, Clinical Modification
MADS: Multimorbidity-Adjusted Disability Score
MDD: major depressive disorder
PR: probability of relevance
THL: Finnish Institute for Health and Welfare
UKB: UK Biobank

## Data Availability

The data sets generated during and analyzed during this study are not publicly available due to patient privacy concerns. The scripts used to compute the Multimorbidity-Adjusted Disability Score are available from the corresponding author upon reasonable request.
