# Supplementary material for: Multicentric Assessment of a Multimorbidity-Adjusted Disability Score to Stratify Depression-Related Risks Using Temporal Disease Maps: Instrument Validation Study
Source: J Med Internet Res. 2024 Jun 24;26:e53162. doi: 10.2196/53162 (PMC11231623; doi:10.2196/53162)
Supplement: Multimedia Appendix 1 [file jmir_v26i1e53162_app1.docx]

**Multicentric validation of a Multimorbidity Adjusted Disability Score to stratify depression-related risks using temporal disease maps.**

Rubèn González-Colom et al.

*(On-line supplementary material)*

Table of content

[Annex 1: Cross-sectional analysis of health outcomes and utilization of healthcare resources 4](#_Toc151585877)

[1.1 CHSS cohort 4](#_Toc151585878)

[Table S1 – Analysis of average number of primary care visits person over the 12 months following MADS assessment itemized by age and sex categories in CHSS. 4](#_Toc151585879)

[Table S2 - Analysis of average number of outpatient care visits person over the 12 months following MADS assessment itemized by age and sex categories in CHSS. 5](#_Toc151585880)

[Table S3 - Analysis of average number of emergency room visits per 100 habitants over the 12 months following MADS assessment itemized by age and sex categories in CHSS. 6](#_Toc151585881)

[Table S4 - Analysis of average number of hospital admissions per 100 habitants over the 12 months following MADS assessment itemized by age and sex categories in CHSS. 7](#_Toc151585882)

[Table S5 - Analysis of average number of mental health visits per 100 habitants over the 12 months following MADS assessment itemized by age and sex categories in CHSS. 8](#_Toc151585883)

[Table S6 - Analysis of average number of different pharmacological prescriptions per person over the 12 months following MADS assessment itemized by age and sex categories in CHSS. 9](#_Toc151585884)

[Table S7 - Analysis of mortality rates per 1000 habitants over the 12 months following MADS assessment itemized by age and sex categories in CHSS. 10](#_Toc151585885)

[Table S8 - Analysis of average pharmacological expenditure per person over the 12 months following MADS assessment itemized by age and sex categories in CHSS. 11](#_Toc151585886)

[Table S9 - Analysis of average hospitalization costs per person over the 12 months following MADS assessment itemized by age and sex categories in CHSS. 12](#_Toc151585887)

[Table S10 - Analysis of average total healthcare expenditure per person over the 12 months following MADS assessment itemized by age and sex categories in CHSS. 13](#_Toc151585888)

[Table S11- Analysis of average number of different antipsychotics prescribed per person over the 12 months following MADS assessment itemized by age and sex categories in CHSS. 14](#_Toc151585889)

[Table S12- Analysis of average number of different anxiolytics prescribed per person over the 12 months following MADS assessment itemized by age and sex categories in CHSS. 15](#_Toc151585890)

[Table S13- Analysis of average number of different hypnotics and sedatives prescribed per person over the 12 months following MADS assessment itemized by age and sex categories in CHSS. 16](#_Toc151585891)

[Table S14- Analysis of average number of different antidepressants prescribed per person over the 12 months following MADS assessment itemized by age and sex categories in CHSS. 17](#_Toc151585892)

[1.2 THL cohort 18](#_Toc151585893)

[Table S15 - Analysis of mortality rates per 1000 habitants over the 12 months following MADS assessment itemized by age and sex categories in THL. 18](#_Toc151585894)

[Table S16 - Analysis of average pharmacological expenditure per person over the 12 months following MADS assessment itemized by age and sex categories in THL. 19](#_Toc151585895)

[Table S18 - Analysis of average hospitalization costs per person over the 12 months following MADS assessment itemized by age and sex categories in THL. 20](#_Toc151585896)

[Table S19- Analysis of average number of different antipsychotics prescribed per person over the 12 months following MADS assessment itemized by age and sex categories in THL. 21](#_Toc151585897)

[Table S20- Analysis of average number of different anxiolytics prescribed per person over the 12 months following MADS assessment itemized by age and sex categories in THL. 22](#_Toc151585898)

[Table S21- Analysis of average number of different hypnotics and sedatives prescribed per person over the 12 months following MADS assessment itemized by age and sex categories in THL. 23](#_Toc151585899)

[Table S22- Analysis of average number of different antidepressants prescribed per person over the 12 months following MADS assessment itemized by age and sex categories in THL. 24](#_Toc151585900)

[1.3 UKB cohort 25](#_Toc151585901)

[Table S23 - Analysis of average number of different pharmacological prescriptions per person over the 12 months following MADS assessment itemized by age and sex categories in UKB. 25](#_Toc151585902)

[Table S24- Analysis of average number of different antipsychotics prescribed per person over the 12 months following MADS assessment itemized by age and sex categories in UKB. 26](#_Toc151585903)

[Table S25- Analysis of average number of different anxiolytics prescribed per person over the 12 months following MADS assessment itemized by age and sex categories in UKB. 27](#_Toc151585904)

[Table S26- Analysis of average number of different hypnotics and sedatives prescribed per person over the 12 months following MADS assessment itemized by age and sex categories in UKB. 28](#_Toc151585905)

[Table S27- Analysis of average number of different antidepressants prescribed per person over the 12 months following MADS assessment itemized by age and sex categories in UKB. 29](#_Toc151585906)

[Annex 2: Longitudinal analysis of disease prevalence and incidence of new disease onsets 30](#_Toc151585907)

[Figure S1 - Longitudinal analysis of disease prevalence and incidence of new onsets of Major Depressive Disorder (ICD-10-CM: F32). 30](#_Toc151585908)

[Figure S2 - Longitudinal analysis of disease prevalence and incidence of new onsets of schizophrenia (ICD-10-CM: F20). 31](#_Toc151585909)

[Figure S3 - Longitudinal analysis of disease prevalence and incidence of new onsets of bipolar disorder (ICD-10-CM: F31). 32](#_Toc151585910)

[Figure S4 - Longitudinal analysis of disease prevalence and incidence of new onsets of anxiety related disorders (ICD-10-CM: F40-41). 33](#_Toc151585911)

[Figure S5 - Longitudinal analysis of disease prevalence and incidence of new onsets of stress related disorders (ICD-10-CM: F43). 34](#_Toc151585912)

[Figure S6 - Longitudinal analysis of disease prevalence and incidence of new onsets of mental disorders related to alcohol abuse (ICD-10-CM: F10). 35](#_Toc151585913)

[Figure S7 - Longitudinal analysis of disease prevalence and incidence of new onsets of irritable bowel syndrome (ICD-10-CM: K58). 36](#_Toc151585914)

[Figure S8 - Longitudinal analysis of disease prevalence and incidence of new onsets of overweight and obesity MDD (ICD-10-CM: E66). 37](#_Toc151585915)

[Figure S9 - Longitudinal analysis of disease prevalence and incidence of new onsets of gastro-oesophageal reflux (ICD-10-CM: K21). 38](#_Toc151585916)

[Annex 3: Adjusted Disability Weights 39](#_Toc151585917)

[Table S28 – Disability weights associated to 88 ICD-10-CM diagnostic codes. 39](#_Toc151585918)

[Annex 4: Probabilities of Relevance 42](#_Toc151585919)

[Table S29 – Probabilities of relevance associated to 88 ICD-10-CM diagnostic codes. 42](#_Toc151585920)

[Annex 5: MADS Pseudocode 56](#_Toc151585921)

# Annex 1: Cross-sectional analysis of health outcomes and utilization of healthcare resources

## CHSS cohort

### Table S1 – Analysis of average number of primary care visits person over the 12 months following MADS assessment itemized by age and sex categories in CHSS.

| MADS  Risk Pyramid Tiers | Primary Care visits  (per person)  Male 0-20 | Primary Care visits  (per person)  Male 20-40 | Primary Care visits  (per person)  Male 40-60 | Primary Care visits  (per person)  Male >60 | Primary Care visits  (per person)  Female 0-20 | Primary Care visits  (per person)  Female 20-40 | Primary Care visits  (per person)  Female 40-60 | Primary Care visits  (per person)  Female >60 |
| --- | --- | --- | --- | --- | --- | --- | --- | --- |
| Very high risk  > P_99_ | 5.38 | 7.4 | 10.49 | 14.61 | 7.76 | 11.18 | 12.83 | 15.58 |
| High risk  (P_95_ – P_99_] | 5.83 | 7.59 | 9.03 | 14.06 | 7.3 | 9.29 | 10.92 | 14.55 |
| Moderate risk  (P_80_ – P_95_] | 5.76 | 5.64 | 7.3 | 11.83 | 6.09 | 6.93 | 8.43 | 11.96 |
| Low risk  (P_50_ – P_80_] | 4.9 | 3.98 | 4.77 | 8.73 | 4.92 | 5.18 | 5.63 | 8.75 |
| Very low risk  ≤ P_50_ | 2.77 | 2.31 | 2.3 | 4.24 | 2.85 | 3.06 | 2.86 | 4.12 |

### Table S2 - Analysis of average number of outpatient care visits person over the 12 months following MADS assessment itemized by age and sex categories in CHSS.

| MADS  Risk Pyramid Tiers | Specialized outpatient visits  (per person)  Male 0-20 | Specialized outpatient visits  (per person)  Male 20-40 | Specialized outpatient visits  (per person)  Male 40-60 | Specialized outpatient visits  (per person)  Male >60 | Specialized outpatient visits  (per person)  Female 0-20 | Specialized outpatient visits  (per person)  Female 20-40 | Specialized outpatient visits  (per person)  Female 40-60 | Specialized outpatient visits  (per person)  Female >60 |
| --- | --- | --- | --- | --- | --- | --- | --- | --- |
| Very high risk  > P_99_ | 1.45 | 2.45 | 3.07 | 3.28 | 3.59 | 2.38 | 3.75 | 3.05 |
| High risk  (P_95_ – P_99_] | 2.33 | 1.28 | 2.46 | 3.06 | 2.2 | 1.65 | 2.83 | 2.75 |
| Moderate risk  (P_80_ – P_95_] | 1.67 | 0.86 | 1.64 | 2.74 | 1.93 | 1.07 | 1.68 | 2.27 |
| Low risk  (P_50_ – P_80_] | 0.97 | 0.56 | 0.88 | 2.12 | 0.83 | 0.73 | 1.09 | 1.76 |
| Very low risk  ≤ P_50_ | 0.43 | 0.28 | 0.34 | 1 | 0.4 | 0.4 | 0.49 | 0.93 |

### Table S3 - Analysis of average number of emergency room visits per 100 habitants over the 12 months following MADS assessment itemized by age and sex categories in CHSS.

| MADS  Risk Pyramid Tiers | Emergency room visits  (visits/100 hab.)  Male 0-20 | Emergency room visits  (visits/100 hab.)  Male 20-40 | Emergency room visits  (visits/100 hab.)  Male 40-60 | Emergency room visits  (visits/100 hab.)  Male >60 | Emergency room visits  (visits/100 hab.)  Female 0-20 | Emergency room visits  (visits/100 hab.)  Female 20-40 | Emergency room visits  (visits/100 hab.)  Female 40-60 | Emergency room visits  (visits/100 hab.)  Female >60 |
| --- | --- | --- | --- | --- | --- | --- | --- | --- |
| Very high risk  > P_99_ | 121.43 | 140.83 | 140.4 | 123.86 | 178.38 | 164.79 | 120.47 | 130.87 |
| High risk  (P_95_ – P_99_] | 76.6 | 94.32 | 77.36 | 91.05 | 128.99 | 102.61 | 70.86 | 91.01 |
| Moderate risk  (P_80_ – P_95_] | 57.12 | 58.73 | 54.11 | 71.87 | 66.76 | 68.81 | 49.56 | 66.8 |
| Low risk  (P_50_ – P_80_] | 52.4 | 39.45 | 31.84 | 46.94 | 48.42 | 52.13 | 32.99 | 42.74 |
| Very low risk  ≤ P_50_ | 28.46 | 25.1 | 17.91 | 22.15 | 25.51 | 31.22 | 17.71 | 19.15 |

### Table S4 - Analysis of average number of hospital admissions per 100 habitants over the 12 months following MADS assessment itemized by age and sex categories in CHSS.

| MADS  Risk Pyramid Tiers | Hospital admissions  (admissions/100 hab.)  Male 0-20 | Hospital admissions  (admissions/100 hab.)  Male 20-40 | Hospital admissions  (admissions/100 hab.)  Male 40-60 | Hospital admissions  (admissions/100 hab.)  Male >60 | Hospital admissions  (admissions/100 hab.)  Female 0-20 | Hospital admissions  (admissions/100 hab.)  Female 20-40 | Hospital admissions  (admissions/100 hab.)  Female 40-60 | Hospital admissions  (admissions/100 hab.)  Female >60 |
| --- | --- | --- | --- | --- | --- | --- | --- | --- |
| Very high risk  > P_99_ | 14.29 | 23.55 | 25.53 | 40.97 | 27.03 | 23.48 | 21.53 | 34.63 |
| High risk  (P_95_ – P_99_] | 7.23 | 8.52 | 16.5 | 35.99 | 15.65 | 9.97 | 12.17 | 26.76 |
| Moderate risk  (P_80_ – P_95_] | 6.16 | 5.07 | 10.49 | 30.61 | 6.62 | 7.66 | 8.35 | 20.8 |
| Low risk  (P_50_ – P_80_] | 3.71 | 3.52 | 6.04 | 20.12 | 2.78 | 6.24 | 5.58 | 14.26 |
| Very low risk  ≤ P_50_ | 1.81 | 1.8 | 2.58 | 8.42 | 1.3 | 3.74 | 2.68 | 5.66 |

### Table S5 - Analysis of average number of mental health visits per 100 habitants over the 12 months following MADS assessment itemized by age and sex categories in CHSS.

| MADS  Risk Pyramid Tiers | Mental Health visits  (visits/100 hab.)  Male 0-20 | Mental Health visits  (visits/100 hab.)  Male 20-40 | Mental Health visits  (visits/100 hab.)  Male 40-60 | Mental Health visits  (visits/100 hab.)  Male >60 | Mental Health visits  (visits/100 hab.)  Female 0-20 | Mental Health visits  (visits/100 hab.)  Female 20-40 | Mental Health visits  (visits/100 hab.)  Female 40-60 | Mental Health visits  (visits/100 hab.)  Female >60 |
| --- | --- | --- | --- | --- | --- | --- | --- | --- |
| Very high risk  > P_99_ | 1430.95 | 923.85 | 706.77 | 318.9 | 818.92 | 584.15 | 654.48 | 278.71 |
| High risk  (P_95_ – P_99_] | 522.98 | 137.33 | 266.19 | 51.6 | 520.58 | 138.01 | 206.16 | 54.03 |
| Moderate risk  (P_80_ – P_95_] | 194.13 | 47.65 | 54.2 | 12.57 | 220.36 | 39.88 | 43.19 | 18.47 |
| Low risk  (P_50_ – P_80_] | 54 | 12.67 | 10.4 | 4.55 | 37.33 | 14.14 | 10.24 | 5.73 |
| Very low risk  ≤ P_50_ | 27.2 | 2.52 | 1.71 | 0.77 | 14.67 | 3.12 | 1.4 | 1.17 |

### Table S6 - Analysis of average number of different pharmacological prescriptions per person over the 12 months following MADS assessment itemized by age and sex categories in CHSS.

| MADS  Risk Pyramid Tiers | Number of prescriptions  (per person)  Male 0-20 | Number of prescriptions  (per person)  Male 20-40 | Number of prescriptions  (per person)  Male 40-60 | Number of prescriptions  (per person)  Male >60 | Number of prescriptions  (per person)  Female 0-20 | Number of prescriptions  (per person)  Female 20-40 | Number of prescriptions  (per person)  Female 40-60 | Number of prescriptions  (per person)  Female >60 |
| --- | --- | --- | --- | --- | --- | --- | --- | --- |
| Very high risk  > P_99_ | 3.19 | 3.99 | 6.87 | 9.85 | 4.85 | 5.61 | 8.32 | 10.79 |
| High risk  (P_95_ – P_99_] | 1.89 | 2.73 | 5.31 | 10.03 | 3.28 | 3.77 | 6.2 | 10.22 |
| Moderate risk  (P_80_ – P_95_] | 1.59 | 1.82 | 3.58 | 8.45 | 1.98 | 2.5 | 4.02 | 8.27 |
| Low risk  (P_50_ – P_80_] | 1.09 | 1.19 | 2.09 | 6.24 | 1.11 | 1.72 | 2.42 | 6.08 |
| Very low risk  ≤ P_50_ | 0.47 | 0.58 | 0.72 | 2.74 | 0.48 | 0.9 | 0.95 | 2.55 |

### Table S7 - Analysis of mortality rates per 1000 habitants over the 12 months following MADS assessment itemized by age and sex categories in CHSS.

| MADS  Risk Pyramid Tiers | Mortality  (cases/1k hab.)  Male 0-20 | Mortality  (cases/1k hab.)  Male 20-40 | Mortality  (cases/1k hab.)  Male 40-60 | Mortality  (cases/1k hab.)  Male >60 | Mortality  (cases/1k hab.)  Female 0-20 | Mortality  (cases/1k hab.)  Female 20-40 | Mortality  (cases/1k hab.)  Female 40-60 | Mortality  (cases/1k hab.)  Female >60 |
| --- | --- | --- | --- | --- | --- | --- | --- | --- |
| Very high risk  > P_99_ | 0 | 4.6 | 12.2 | 131 | 0 | 0 | 9.7 | 96.5 |
| High risk  (P_95_ – P_99_] | 0 | 3.2 | 12.3 | 104.8 | 0 | 0 | 4.9 | 70.4 |
| Moderate risk  (P_80_ – P_95_] | 1.1 | 0.6 | 4.7 | 73.3 | 1.2 | 0.2 | 2 | 52.8 |
| Low risk  (P_50_ – P_80_] | 0 | 0.5 | 2.2 | 36.7 | 0 | 0.1 | 1.2 | 29.8 |
| Very low risk  ≤ P_50_ | 0.1 | 0.2 | 1.1 | 14.4 | 0 | 0.1 | 0.8 | 9.3 |

### Table S8 - Analysis of average pharmacological expenditure per person over the 12 months following MADS assessment itemized by age and sex categories in CHSS.

| MADS  Risk Pyramid Tiers | Medication expenditure in €  (per person)  Male 0-20 | Medication expenditure in €  (per person)  Male 20-40 | Medication expenditure in €  (per person)  Male 40-60 | Medication expenditure in €  (per person)  Male >60 | Medication expenditure in €  (per person)  Female 0-20 | Medication expenditure in €  (per person)  Female 20-40 | Medication expenditure in €  (per person)  Female 40-60 | Medication expenditure in €  (per person)  Female >60 |
| --- | --- | --- | --- | --- | --- | --- | --- | --- |
| Very high risk  > P_99_ | 390 | 1,279 | 1,552 | 1,525 | 464 | 590 | 1,166 | 1,200 |
| High risk  (P_95_ – P_99_] | 350 | 256 | 831 | 1,193 | 160 | 234 | 709 | 919 |
| Moderate risk  (P_80_ – P_95_] | 126 | 148 | 358 | 1,029 | 117 | 153 | 353 | 725 |
| Low risk  (P_50_ – P_80_] | 46 | 85 | 201 | 749 | 34 | 106 | 222 | 510 |
| Very low risk  ≤ P_50_ | 25 | 43 | 77 | 364 | 11 | 41 | 98 | 246 |

### Table S9 - Analysis of average hospitalization costs per person over the 12 months following MADS assessment itemized by age and sex categories in CHSS.

| MADS  Risk Pyramid Tiers | Hospitalization expenditure in €  (per person)  Male 0-20 | Hospitalization expenditure in €  (per person)  Male 20-40 | Hospitalization expenditure in €  (per person)  Male 40-60 | Hospitalization expenditure in €  (per person)  Male >60 | Hospitalization expenditure in €  (per person)  Female 0-20 | Hospitalization expenditure in €  (per person)  Female 20-40 | Hospitalization expenditure in €  (per person)  Female 40-60 | Hospitalization expenditure in €  (per person)  Female >60 |
| --- | --- | --- | --- | --- | --- | --- | --- | --- |
| Very high risk  > P_99_ | 271 | 466 | 496 | 777 | 489 | 426 | 407 | 645 |
| High risk  (P_95_ – P_99_] | 124 | 156 | 317 | 679 | 298 | 175 | 229 | 496 |
| Moderate risk  (P_80_ – P_95_] | 114 | 93 | 200 | 583 | 122 | 131 | 155 | 388 |
| Low risk  (P_50_ – P_80_] | 66 | 65 | 114 | 385 | 51 | 107 | 103 | 267 |
| Very low risk  ≤ P_50_ | 33 | 32 | 48 | 164 | 23 | 63 | 49 | 107 |

### Table S10 - Analysis of average total healthcare expenditure per person over the 12 months following MADS assessment itemized by age and sex categories in CHSS.

| MADS  Risk Pyramid Tiers | Total expenditure in €  (per person)  Male 0-20 | Total expenditure in €  (per person)  Male 20-40 | Total expenditure in €  (per person)  Male 40-60 | Total expenditure in €  (per person)  Male >60 | Total expenditure in €  (per person)  Female 0-20 | Total expenditure in €  (per person)  Female 20-40 | Total expenditure in €  (per person)  Female 40-60 | Total expenditure in €  (per person)  Female >60 |
| --- | --- | --- | --- | --- | --- | --- | --- | --- |
| Very high risk  > P_99_ | 6,051 | 10,145 | 12,235 | 17,674 | 10,242 | 7,439 | 11,028 | 14,934 |
| High risk  (P_95_ – P_99_] | 3,532 | 3,431 | 8,477 | 15,225 | 4,669 | 3,256 | 5,776 | 10,047 |
| Moderate risk  (P_80_ – P_95_] | 2,224 | 2,014 | 4,784 | 11,387 | 2,782 | 2,036 | 2,987 | 7,021 |
| Low risk  (P_50_ – P_80_] | 1,350 | 1,308 | 2,677 | 7,392 | 1,191 | 1,517 | 1,822 | 4,513 |
| Very low risk  ≤ P_50_ | 813 | 757 | 1,039 | 3,475 | 721 | 929 | 910 | 1,776 |

### Table S11- Analysis of average number of different antipsychotics prescribed per person over the 12 months following MADS assessment itemized by age and sex categories in CHSS.

| MADS  Risk Pyramid Tiers | Antipsychotic  (N05A)  (per person)  Male 0-20 | Antipsychotic  (N05A)  (per person)  Male 20-40 | Antipsychotic  (N05A)  (per person)  Male 40-60 | Antipsychotic  (N05A)  (per person)  Male >60 | Antipsychotic  (N05A)  (per person)  Female 0-20 | Antipsychotic  (N05A)  (per person)  Female 20-40 | Antipsychotic  (N05A)  (per person)  Female 40-60 | Antipsychotic  (N05A)  (per person)  Female >60 |
| --- | --- | --- | --- | --- | --- | --- | --- | --- |
| Very high risk  > P_99_ | 1.23 | 1.57 | 1.65 | 1.08 | 0.87 | 0.88 | 0.95 | 0.68 |
| High risk  (P_95_ – P_99_] | 0.74 | 0.27 | 0.73 | 0.20 | 0.33 | 0.18 | 0.28 | 0.17 |
| Moderate risk  (P_80_ – P_95_] | 0.41 | 0.24 | 0.18 | 0.14 | 0.26 | 0.13 | 0.08 | 0.12 |
| Low risk  (P_50_ – P_80_] | 0.17 | 0.15 | 0.16 | 0.09 | 0.09 | 0.07 | 0.08 | 0.09 |
| Very low risk  ≤ P_50_ | 0.10 | 0.09 | 0.09 | 0.06 | 0.06 | 0.06 | 0.06 | 0.05 |

### Table S12- Analysis of average number of different anxiolytics prescribed per person over the 12 months following MADS assessment itemized by age and sex categories in CHSS.

| MADS  Risk Pyramid Tiers | Anxiolytic  (N05B)  (per person)  Male 0-20 | Anxiolytic  (N05B)  (per person)  Male 20-40 | Anxiolytic  (N05B)  (per person)  Male 40-60 | Anxiolytic  (N05B)  (per person)  Male >60 | Anxiolytic  (N05B)  (per person)  Female 0-20 | Anxiolytic  (N05B)  (per person)  Female 20-40 | Anxiolytic  (N05B)  (per person)  Female 40-60 | Anxiolytic  (N05B)  (per person)  Female >60 |
| --- | --- | --- | --- | --- | --- | --- | --- | --- |
| Very high risk  > P_99_ | 0.24 | 0.37 | 0.74 | 0.45 | 0.18 | 0.41 | 0.72 | 0.67 |
| High risk  (P_95_ – P_99_] | 0.13 | 0.30 | 0.58 | 0.52 | 0.14 | 0.32 | 0.63 | 0.64 |
| Moderate risk  (P_80_ – P_95_] | 0.19 | 0.29 | 0.47 | 0.49 | 0.15 | 0.26 | 0.47 | 0.58 |
| Low risk  (P_50_ – P_80_] | 0.06 | 0.25 | 0.37 | 0.47 | 0.09 | 0.22 | 0.34 | 0.51 |
| Very low risk  ≤ P_50_ | 0.04 | 0.11 | 0.24 | 0.38 | 0.07 | 0.16 | 0.25 | 0.40 |

### Table S13- Analysis of average number of different hypnotics and sedatives prescribed per person over the 12 months following MADS assessment itemized by age and sex categories in CHSS.

| MADS  Risk Pyramid Tiers | Hypnotics and sedatives  (N05C)  (per person)  Male 0-20 | Hypnotics and sedatives  (N05C)  (per person)  Male 20-40 | Hypnotics and sedatives  (N05C)  (per person)  Male 40-60 | Hypnotics and sedatives  (N05C)  (per person)  Male >60 | Hypnotics and sedatives  (N05C)  (per person)  Female 0-20 | Hypnotics and sedatives  (N05C)  (per person)  Female 20-40 | Hypnotics and sedatives  (N05C)  (per person)  Female 40-60 | Hypnotics and sedatives  (N05C)  (per person)  Female >60 |
| --- | --- | --- | --- | --- | --- | --- | --- | --- |
| Very high risk  > P_99_ | 0.10 | 0.11 | 0.20 | 0.19 | 0.01 | 0.18 | 0.25 | 0.28 |
| High risk  (P_95_ – P_99_] | 0.01 | 0.08 | 0.14 | 0.20 | 0.05 | 0.05 | 0.16 | 0.21 |
| Moderate risk  (P_80_ – P_95_] | 0.02 | 0.05 | 0.10 | 0.16 | 0.04 | 0.04 | 0.10 | 0.18 |
| Low risk  (P_50_ – P_80_] | 0.00 | 0.03 | 0.09 | 0.15 | 0.01 | 0.03 | 0.07 | 0.17 |
| Very low risk  ≤ P_50_ | 0.00 | 0.03 | 0.07 | 0.13 | 0.00 | 0.02 | 0.05 | 0.14 |

### Table S14- Analysis of average number of different antidepressants prescribed per person over the 12 months following MADS assessment itemized by age and sex categories in CHSS.

| MADS  Risk Pyramid Tiers | Antidepressant  (N06A)  (per person)  Male 0-20 | Antidepressant  (N06A)  (per person)  Male 20-40 | Antidepressant  (N06A)  (per person)  Male 40-60 | Antidepressant  (N06A)  (per person)  Male >60 | Antidepressant  (N06A)  (per person)  Female 0-20 | Antidepressant  (N06A)  (per person)  Female 20-40 | Antidepressant  (N06A)  (per person)  Female 40-60 | Antidepressant  (N06A)  (per person)  Female >60 |
| --- | --- | --- | --- | --- | --- | --- | --- | --- |
| Very high risk  > P_99_ | 0.64 | 0.52 | 0.90 | 0.80 | 0.91 | 0.76 | 1.21 | 1.11 |
| High risk  (P_95_ – P_99_] | 0.46 | 0.56 | 0.78 | 0.77 | 0.64 | 0.63 | 0.91 | 0.94 |
| Moderate risk  (P_80_ – P_95_] | 0.20 | 0.42 | 0.52 | 0.46 | 0.31 | 0.45 | 0.57 | 0.62 |
| Low risk  (P_50_ – P_80_] | 0.05 | 0.27 | 0.32 | 0.22 | 0.10 | 0.27 | 0.34 | 0.34 |
| Very low risk  ≤ P_50_ | 0.05 | 0.15 | 0.16 | 0.16 | 0.08 | 0.18 | 0.17 | 0.22 |

## THL cohort

### Table S15 - Analysis of mortality rates per 1000 habitants over the 12 months following MADS assessment itemized by age and sex categories in THL.

| MADS  Risk Pyramid Tiers | Mortality  (cases/1k hab.)  Male 0-20 | Mortality  (cases/1k hab.)  Male 20-40 | Mortality  (cases/1k hab.)  Male 40-60 | Mortality  (cases/1k hab.)  Male >60 | Mortality  (cases/1k hab.)  Female 0-20 | Mortality  (cases/1k hab.)  Female 20-40 | Mortality  (cases/1k hab.)  Female 40-60 | Mortality  (cases/1k hab.)  Female >60 |
| --- | --- | --- | --- | --- | --- | --- | --- | --- |
| Very high risk  > P_99_ | N.A. | 0.00 | 50.00 | 55.00 | N.A. | 0.00 | 0.00 | 39.00 |
| High risk  (P_95_ – P_99_] | N.A. | 0.00 | 0.00 | 78.00 | N.A. | 0.00 | 9.00 | 40.00 |
| Moderate risk  (P_80_ – P_95_] | N.A. | 0.00 | 9.00 | 56.00 | N.A. | 0.00 | 5.00 | 35.00 |
| Low risk  (P_50_ – P_80_] | N.A. | 0.00 | 5.00 | 27.00 | N.A. | 0.00 | 3.00 | 17.00 |
| Very low risk  ≤ P_50_ | N.A. | 2.00 | 3.00 | 14.00 | N.A. | 0.00 | 1.00 | 9.00 |

### Table S16 - Analysis of average pharmacological expenditure per person over the 12 months following MADS assessment itemized by age and sex categories in THL.

| MADS  Risk Pyramid Tiers | Medication expenditure in €  (per person)  Male 0-20 | Medication expenditure in €  (per person)  Male 20-40 | Medication expenditure in €  (per person)  Male 40-60 | Medication expenditure in €  (per person)  Male >60 | Medication expenditure in €  (per person)  Female 0-20 | Medication expenditure in €  (per person)  Female 20-40 | Medication expenditure in €  (per person)  Female 40-60 | Medication expenditure in €  (per person)  Female >60 |
| --- | --- | --- | --- | --- | --- | --- | --- | --- |
| Very high risk  > P_99_ | N.A. | 277 | 1,012 | 1,013 | N.A. | 501 | 601 | 1,125 |
| High risk  (P_95_ – P_99_] | N.A. | 1,550 | 864 | 1,177 | N.A. | 482 | 1,074 | 1,344 |
| Moderate risk  (P_80_ – P_95_] | N.A. | 863 | 925 | 1,349 | N.A. | 313 | 733 | 1,123 |
| Low risk  (P_50_ – P_80_] | N.A. | 169 | 588 | 1,079 | N.A. | 219 | 608 | 879 |
| Very low risk  ≤ P_50_ | N.A. | 84 | 182 | 564 | N.A. | 148 | 207 | 435 |

### Table S18 - Analysis of average hospitalization costs per person over the 12 months following MADS assessment itemized by age and sex categories in THL.

| MADS  Risk Pyramid Tiers | Hospitalization expenditure in €  (per person)  Male 0-20 | Hospitalization expenditure in €  (per person)  Male 20-40 | Hospitalization expenditure in €  (per person)  Male 40-60 | Hospitalization expenditure in €  (per person)  Male >60 | Hospitalization expenditure in €  (per person)  Female 0-20 | Hospitalization expenditure in €  (per person)  Female 20-40 | Hospitalization expenditure in €  (per person)  Female 40-60 | Hospitalization expenditure in €  (per person)  Female >60 |
| --- | --- | --- | --- | --- | --- | --- | --- | --- |
| Very high risk  > P_99_ | N.A. | 198 | 139 | 412 | N.A. | 305 | 272 | 221 |
| High risk  (P_95_ – P_99_] | N.A. | 837 | 247 | 308 | N.A. | 716 | 417 | 255 |
| Moderate risk  (P_80_ – P_95_] | N.A. | 245 | 90 | 369 | N.A. | 160 | 193 | 237 |
| Low risk  (P_50_ – P_80_] | N.A. | 19 | 94 | 207 | N.A. | 174 | 160 | 227 |
| Very low risk  ≤ P_50_ | N.A. | 28 | 115 | 160 | N.A. | 71 | 105 | 121 |

### Table S19- Analysis of average number of different antipsychotics prescribed per person over the 12 months following MADS assessment itemized by age and sex categories in THL.

| MADS  Risk Pyramid Tiers | Antipsychotic  (N05A)  (per person)  Male 0-20 | Antipsychotic  (N05A)  (per person)  Male 20-40 | Antipsychotic  (N05A)  (per person)  Male 40-60 | Antipsychotic  (N05A)  (per person)  Male >60 | Antipsychotic  (N05A)  (per person)  Female 0-20 | Antipsychotic  (N05A)  (per person)  Female 20-40 | Antipsychotic  (N05A)  (per person)  Female 40-60 | Antipsychotic  (N05A)  (per person)  Female >60 |
| --- | --- | --- | --- | --- | --- | --- | --- | --- |
| Very high risk  > P_99_ | N.A. | 0.63 | 0.70 | 0.58 | N.A. | 0.38 | 0.52 | 0.62 |
| High risk  (P_95_ – P_99_] | N.A. | 0.06 | 0.28 | 0.27 | N.A. | 0.11 | 0.30 | 0.28 |
| Moderate risk  (P_80_ – P_95_] | N.A. | 0.06 | 0.07 | 0.09 | N.A. | 0.06 | 0.07 | 0.09 |
| Low risk  (P_50_ – P_80_] | N.A. | 0.00 | 0.03 | 0.03 | N.A. | 0.02 | 0.02 | 0.04 |
| Very low risk  ≤ P_50_ | N.A. | 0.00 | 0.01 | 0.02 | N.A. | 0.01 | 0.01 | 0.02 |

### Table S20- Analysis of average number of different anxiolytics prescribed per person over the 12 months following MADS assessment itemized by age and sex categories in THL.

| MADS  Risk Pyramid Tiers | Anxiolytic  (N05B)  (per person)  Male 0-20 | Anxiolytic  (N05B)  (per person)  Male 20-40 | Anxiolytic  (N05B)  (per person)  Male 40-60 | Anxiolytic  (N05B)  (per person)  Male >60 | Anxiolytic  (N05B)  (per person)  Female 0-20 | Anxiolytic  (N05B)  (per person)  Female 20-40 | Anxiolytic  (N05B)  (per person)  Female 40-60 | Anxiolytic  (N05B)  (per person)  Female >60 |
| --- | --- | --- | --- | --- | --- | --- | --- | --- |
| Very high risk  > P_99_ | N.A. | 0.13 | 0.30 | 0.20 | N.A. | 0.13 | 0.24 | 0.20 |
| High risk  (P_95_ – P_99_] | N.A. | 0.19 | 0.16 | 0.16 | N.A. | 0.22 | 0.21 | 0.20 |
| Moderate risk  (P_80_ – P_95_] | N.A. | 0.04 | 0.06 | 0.08 | N.A. | 0.07 | 0.08 | 0.10 |
| Low risk  (P_50_ – P_80_] | N.A. | 0.02 | 0.02 | 0.05 | N.A. | 0.04 | 0.03 | 0.06 |
| Very low risk  ≤ P_50_ | N.A. | 0.01 | 0.01 | 0.02 | N.A. | 0.02 | 0.02 | 0.03 |

### Table S21- Analysis of average number of different hypnotics and sedatives prescribed per person over the 12 months following MADS assessment itemized by age and sex categories in THL.

| MADS  Risk Pyramid Tiers | Hypnotics and sedatives  (N05C)  (per person)  Male 0-20 | Hypnotics and sedatives  (N05C)  (per person)  Male 20-40 | Hypnotics and sedatives  (N05C)  (per person)  Male 40-60 | Hypnotics and sedatives  (N05C)  (per person)  Male >60 | Hypnotics and sedatives  (N05C)  (per person)  Female 0-20 | Hypnotics and sedatives  (N05C)  (per person)  Female 20-40 | Hypnotics and sedatives  (N05C)  (per person)  Female 40-60 | Hypnotics and sedatives  (N05C)  (per person)  Female >60 |
| --- | --- | --- | --- | --- | --- | --- | --- | --- |
| Very high risk  > P_99_ | N.A. | 0.00 | 0.10 | 0.09 | N.A. | 0.25 | 0.10 | 0.19 |
| High risk  (P_95_ – P_99_] | N.A. | 0.06 | 0.10 | 0.10 | N.A. | 0.07 | 0.14 | 0.15 |
| Moderate risk  (P_80_ – P_95_] | N.A. | 0.03 | 0.04 | 0.10 | N.A. | 0.03 | 0.10 | 0.13 |
| Low risk  (P_50_ – P_80_] | N.A. | 0.01 | 0.04 | 0.06 | N.A. | 0.03 | 0.04 | 0.10 |
| Very low risk  ≤ P_50_ | N.A. | 0.01 | 0.03 | 0.04 | N.A. | 0.00 | 0.03 | 0.07 |

### Table S22- Analysis of average number of different antidepressants prescribed per person over the 12 months following MADS assessment itemized by age and sex categories in THL.

| MADS  Risk Pyramid Tiers | Antidepressant  (N06A)  (per person)  Male 0-20 | Antidepressant  (N06A)  (per person)  Male 20-40 | Antidepressant  (N06A)  (per person)  Male 40-60 | Antidepressant  (N06A)  (per person)  Male >60 | Antidepressant  (N06A)  (per person)  Female 0-20 | Antidepressant  (N06A)  (per person)  Female 20-40 | Antidepressant  (N06A)  (per person)  Female 40-60 | Antidepressant  (N06A)  (per person)  Female >60 |
| --- | --- | --- | --- | --- | --- | --- | --- | --- |
| Very high risk  > P_99_ | N.A. | 0.38 | 0.50 | 0.42 | N.A. | 0.75 | 0.52 | 0.37 |
| High risk  (P_95_ – P_99_] | N.A. | 0.44 | 0.44 | 0.33 | N.A. | 0.39 | 0.49 | 0.41 |
| Moderate risk  (P_80_ – P_95_] | N.A. | 0.22 | 0.23 | 0.22 | N.A. | 0.22 | 0.38 | 0.29 |
| Low risk  (P_50_ – P_80_] | N.A. | 0.07 | 0.08 | 0.09 | N.A. | 0.11 | 0.13 | 0.13 |
| Very low risk  ≤ P_50_ | N.A. | 0.04 | 0.04 | 0.04 | N.A. | 0.06 | 0.07 | 0.08 |

## UKB cohort

### Table S23 - Analysis of average number of different pharmacological prescriptions per person over the 12 months following MADS assessment itemized by age and sex categories in UKB.

| MADS  Risk Pyramid Tiers | Number of prescriptions  (per person)  Male 0-20 | Number of prescriptions  (per person)  Male 20-40 | Number of prescriptions  (per person)  Male 40-60 | Number of prescriptions  (per person)  Male >60 | Number of prescriptions  (per person)  Female 0-20 | Number of prescriptions  (per person)  Female 20-40 | Number of prescriptions  (per person)  Female 40-60 | Number of prescriptions  (per person)  Female >60 |
| --- | --- | --- | --- | --- | --- | --- | --- | --- |
| Very high risk  > P_99_ | N.A. | N.A. | 13.63 | 13.43 | N.A. | N.A. | 13.63 | 13.43 |
| High risk  (P_95_ – P_99_] | N.A. | N.A. | 11.91 | 12.20 | N.A. | N.A. | 11.91 | 12.20 |
| Moderate risk  (P_80_ – P_95_] | N.A. | N.A. | 10.76 | 12.29 | N.A. | N.A. | 10.76 | 12.29 |
| Low risk  (P_50_ – P_80_] | N.A. | N.A. | 9.18 | 11.65 | N.A. | N.A. | 9.18 | 11.65 |
| Very low risk  ≤ P_50_ | N.A. | N.A. | 7.11 | 9.88 | N.A. | N.A. | 7.11 | 9.88 |

### Table S24- Analysis of average number of different antipsychotics prescribed per person over the 12 months following MADS assessment itemized by age and sex categories in UKB.

| MADS  Risk Pyramid Tiers | Antipsychotic  (N05A)  (per person)  Male 0-20 | Antipsychotic  (N05A)  (per person)  Male 20-40 | Antipsychotic  (N05A)  (per person)  Male 40-60 | Antipsychotic  (N05A)  (per person)  Male >60 | Antipsychotic  (N05A)  (per person)  Female 0-20 | Antipsychotic  (N05A)  (per person)  Female 20-40 | Antipsychotic  (N05A)  (per person)  Female 40-60 | Antipsychotic  (N05A)  (per person)  Female >60 |
| --- | --- | --- | --- | --- | --- | --- | --- | --- |
| Very high risk  > P_99_ | N.A. | N.A. | 0.43 | 0.23 | N.A. | N.A. | 0.37 | 0.32 |
| High risk  (P_95_ – P_99_] | N.A. | N.A. | 0.13 | 0.17 | N.A. | N.A. | 0.21 | 0.17 |
| Moderate risk  (P_80_ – P_95_] | N.A. | N.A. | 0.10 | 0.14 | N.A. | N.A. | 0.17 | 0.17 |
| Low risk  (P_50_ – P_80_] | N.A. | N.A. | 0.07 | 0.12 | N.A. | N.A. | 0.12 | 0.17 |
| Very low risk  ≤ P_50_ | N.A. | N.A. | 0.05 | 0.11 | N.A. | N.A. | 0.12 | 0.14 |

### Table S25- Analysis of average number of different anxiolytics prescribed per person over the 12 months following MADS assessment itemized by age and sex categories in UKB.

| MADS  Risk Pyramid Tiers | Anxiolytic  (N05B)  (per person)  Male 0-20 | Anxiolytic  (N05B)  (per person)  Male 20-40 | Anxiolytic  (N05B)  (per person)  Male 40-60 | Anxiolytic  (N05B)  (per person)  Male >60 | Anxiolytic  (N05B)  (per person)  Female 0-20 | Anxiolytic  (N05B)  (per person)  Female 20-40 | Anxiolytic  (N05B)  (per person)  Female 40-60 | Anxiolytic  (N05B)  (per person)  Female >60 |
| --- | --- | --- | --- | --- | --- | --- | --- | --- |
| Very high risk  > P_99_ | N.A. | N.A. | 0.23 | 0.24 | N.A. | N.A. | 0.33 | 0.26 |
| High risk  (P_95_ – P_99_] | N.A. | N.A. | 0.19 | 0.20 | N.A. | N.A. | 0.20 | 0.19 |
| Moderate risk  (P_80_ – P_95_] | N.A. | N.A. | 0.16 | 0.16 | N.A. | N.A. | 0.17 | 0.15 |
| Low risk  (P_50_ – P_80_] | N.A. | N.A. | 0.09 | 0.13 | N.A. | N.A. | 0.13 | 0.13 |
| Very low risk  ≤ P_50_ | N.A. | N.A. | 0.10 | 0.08 | N.A. | N.A. | 0.09 | 0.10 |

### Table S26- Analysis of average number of different hypnotics and sedatives prescribed per person over the 12 months following MADS assessment itemized by age and sex categories in UKB.

| MADS  Risk Pyramid Tiers | Hypnotics and sedatives  (N05C)  (per person)  Male 0-20 | Hypnotics and sedatives  (N05C)  (per person)  Male 20-40 | Hypnotics and sedatives  (N05C)  (per person)  Male 40-60 | Hypnotics and sedatives  (N05C)  (per person)  Male >60 | Hypnotics and sedatives  (N05C)  (per person)  Female 0-20 | Hypnotics and sedatives  (N05C)  (per person)  Female 20-40 | Hypnotics and sedatives  (N05C)  (per person)  Female 40-60 | Hypnotics and sedatives  (N05C)  (per person)  Female >60 |
| --- | --- | --- | --- | --- | --- | --- | --- | --- |
| Very high risk  > P_99_ | N.A. | N.A. | 0.18 | 0.27 | N.A. | N.A. | 0.21 | 0.29 |
| High risk  (P_95_ – P_99_] | N.A. | N.A. | 0.16 | 0.21 | N.A. | N.A. | 0.17 | 0.20 |
| Moderate risk  (P_80_ – P_95_] | N.A. | N.A. | 0.17 | 0.21 | N.A. | N.A. | 0.16 | 0.16 |
| Low risk  (P_50_ – P_80_] | N.A. | N.A. | 0.10 | 0.18 | N.A. | N.A. | 0.11 | 0.12 |
| Very low risk  ≤ P_50_ | N.A. | N.A. | 0.08 | 0.10 | N.A. | N.A. | 0.09 | 0.11 |

### Table S27- Analysis of average number of different antidepressants prescribed per person over the 12 months following MADS assessment itemized by age and sex categories in UKB.

| MADS  Risk Pyramid Tiers | Antidepressant  (N06A)  (per person)  Male 0-20 | Antidepressant  (N06A)  (per person)  Male 20-40 | Antidepressant  (N06A)  (per person)  Male 40-60 | Antidepressant  (N06A)  (per person)  Male >60 | Antidepressant  (N06A)  (per person)  Female 0-20 | Antidepressant  (N06A)  (per person)  Female 20-40 | Antidepressant  (N06A)  (per person)  Female 40-60 | Antidepressant  (N06A)  (per person)  Female >60 |
| --- | --- | --- | --- | --- | --- | --- | --- | --- |
| Very high risk  > P_99_ | N.A. | N.A. | 1.07 | 0.61 | N.A. | N.A. | 0.82 | 0.77 |
| High risk  (P_95_ – P_99_] | N.A. | N.A. | 0.72 | 0.64 | N.A. | N.A. | 0.86 | 0.63 |
| Moderate risk  (P_80_ – P_95_] | N.A. | N.A. | 0.59 | 0.42 | N.A. | N.A. | 0.68 | 0.51 |
| Low risk  (P_50_ – P_80_] | N.A. | N.A. | 0.31 | 0.29 | N.A. | N.A. | 0.49 | 0.37 |
| Very low risk  ≤ P_50_ | N.A. | N.A. | 0.22 | 0.22 | N.A. | N.A. | 0.33 | 0.28 |

# Annex 2: Longitudinal analysis of disease prevalence and incidence of new disease onsets


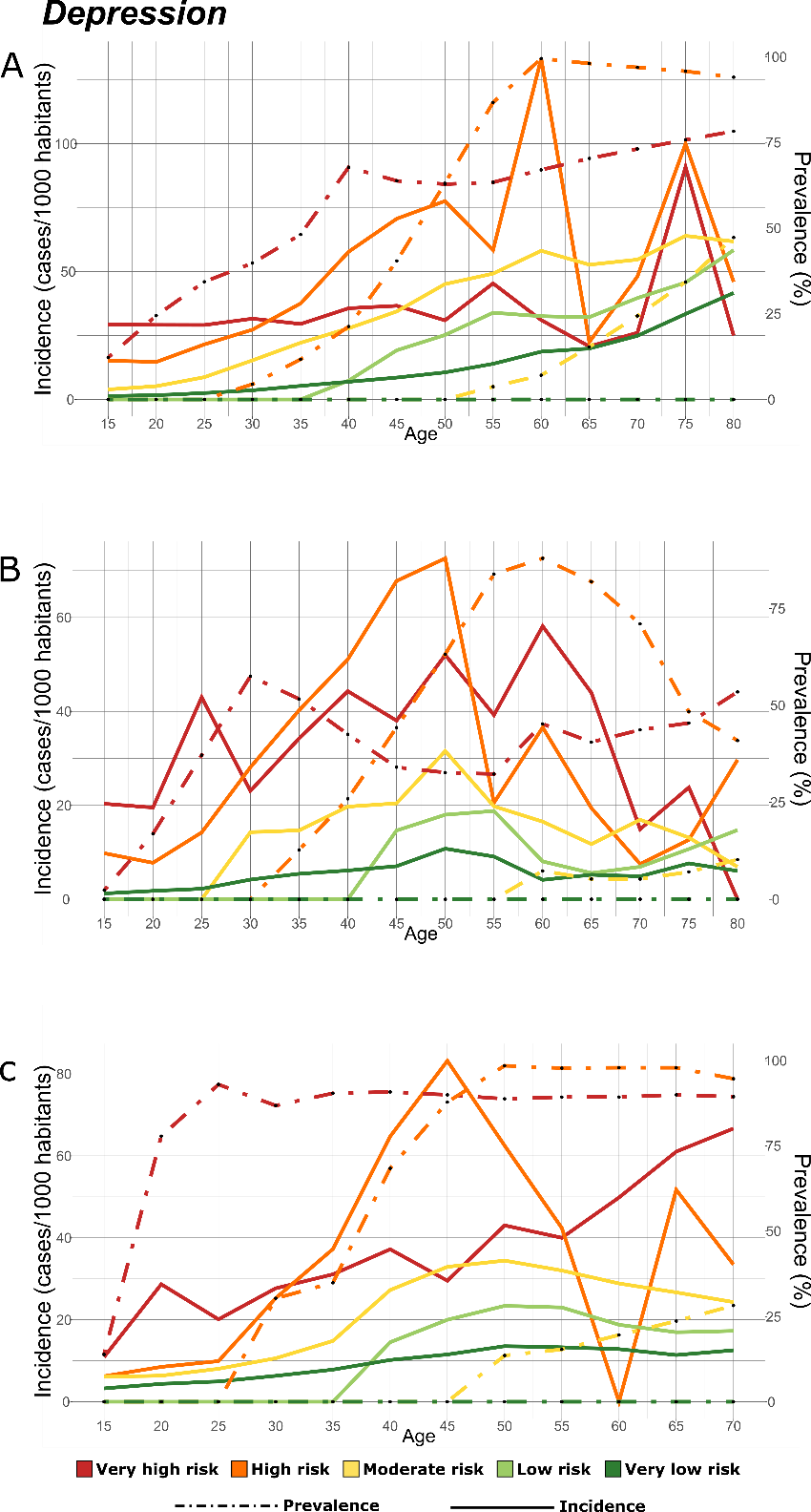


Figure S1 - Longitudinal analysis of disease prevalence and incidence of new onsets of Major Depressive Disorder (ICD-10-CM: F32). *Panel A) CHSS cohort; Panel B) THL cohort; Panel C)UKB cohort. Disease incidence is assessed in a 5-years interval, plotted in the left y-axis and represented with solid lines. Disease prevalence is plotted in the right y-axis and represented with dashed lines. The line colours correspond to the MADS risk pyramid tiers: red: very high-risk group; orange: high-risk group; yellow: medium-risk group; light green: low-risk group; green: very low-risk group.*

***
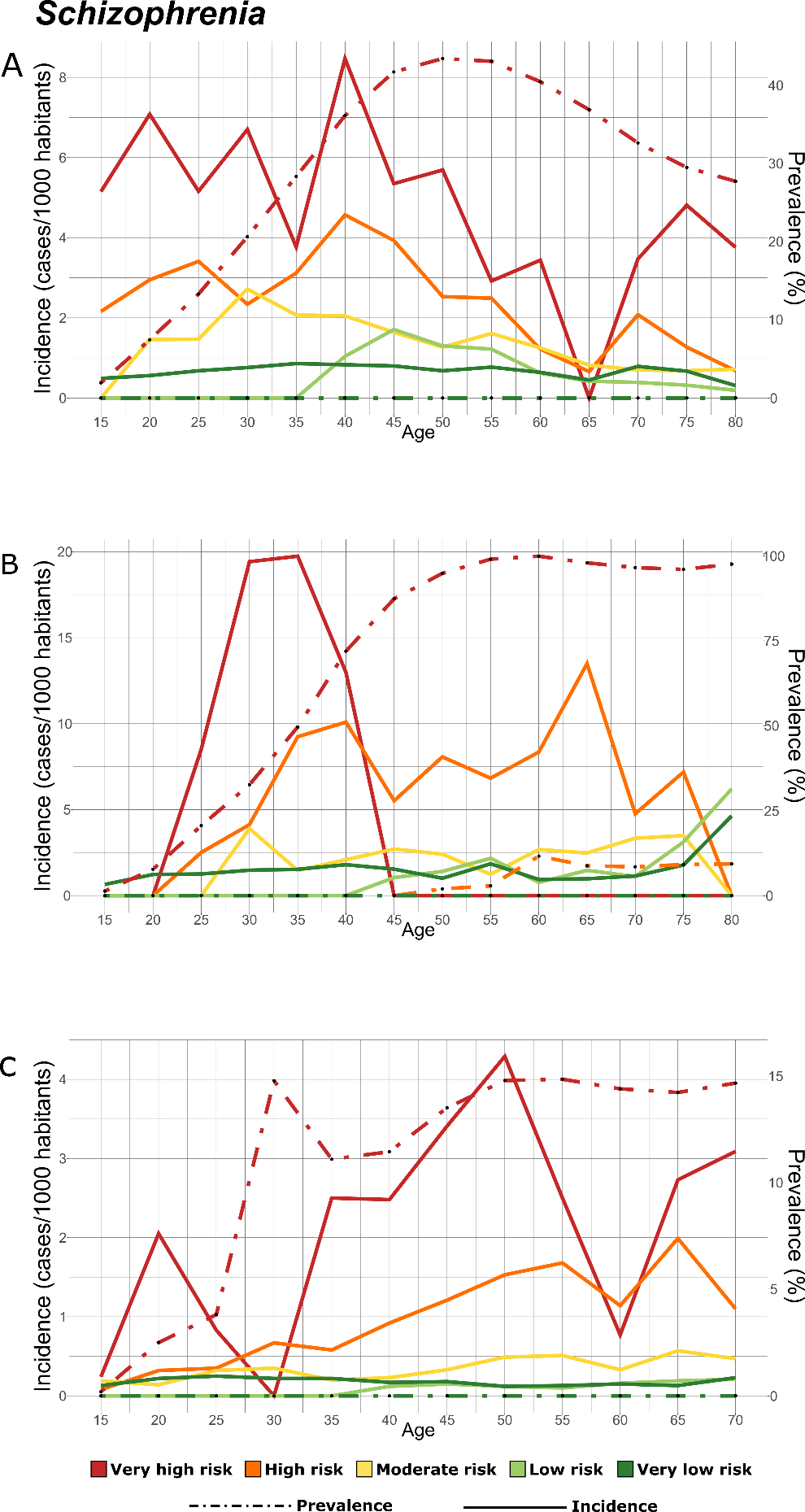
***

Figure S2 - Longitudinal analysis of disease prevalence and incidence of new onsets of schizophrenia (ICD-10-CM: F20). *Panel A) CHSS cohort; Panel B) THL cohort; Panel C)UKB cohort. Disease incidence is assessed in a 5-years interval, plotted in the left y-axis and represented with solid lines. Disease prevalence is plotted in the right y-axis and represented with dashed lines. The line colours correspond to the MADS risk pyramid tiers: red: very high-risk group; orange: high-risk group; yellow: medium-risk group; light green: low-risk group; green: very low-risk group.*

*
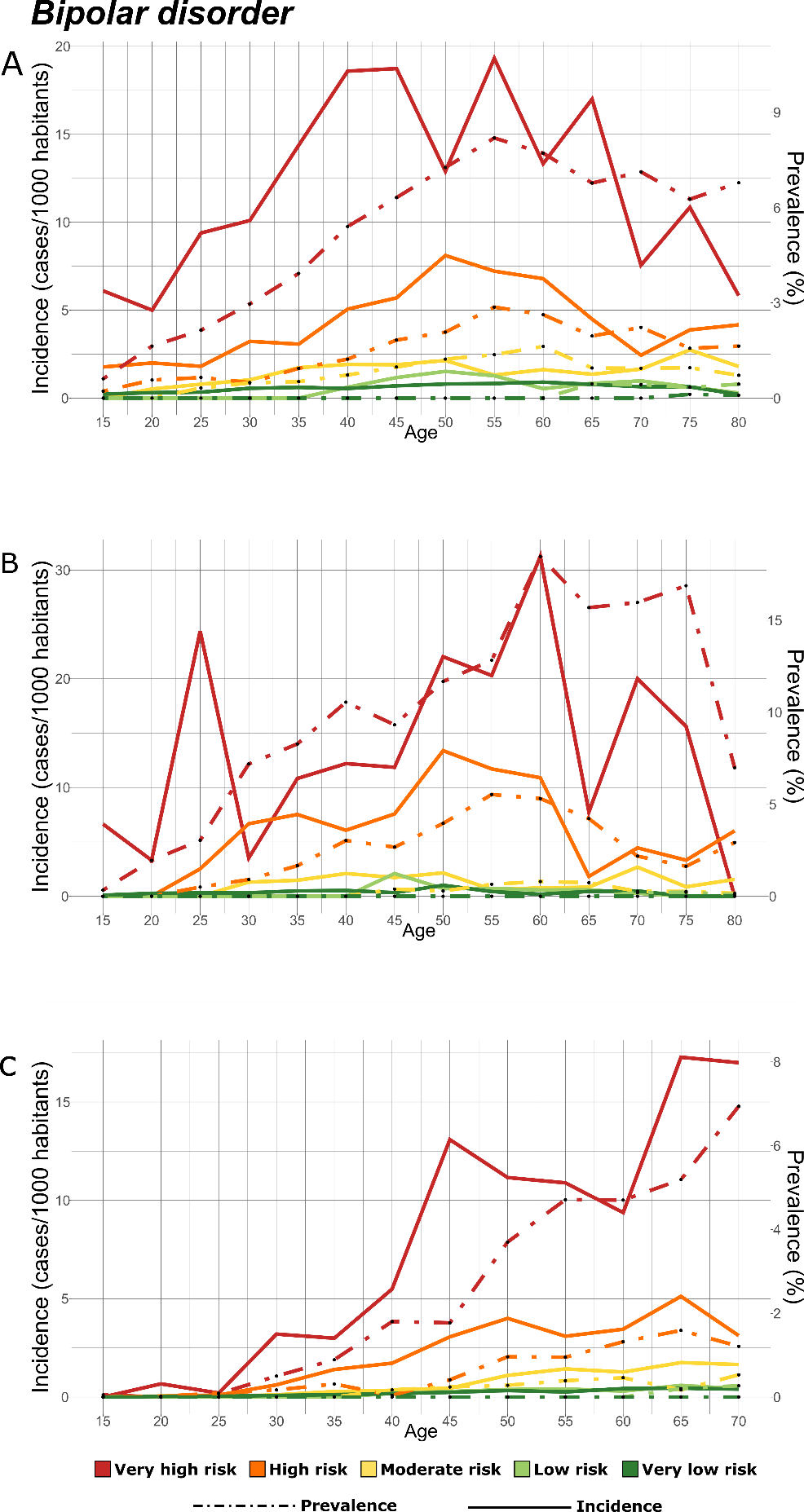
*

Figure S3 - Longitudinal analysis of disease prevalence and incidence of new onsets of bipolar disorder (ICD-10-CM: F31). *Panel A) CHSS cohort; Panel B) THL cohort; Panel C)UKB cohort. Disease incidence is assessed in a 5-years interval, plotted in the left y-axis and represented with solid lines. Disease prevalence is plotted in the right y-axis and represented with dashed lines. The line colours correspond to the MADS risk pyramid tiers: red: very high-risk group; orange: high-risk group; yellow: medium-risk group; light green: low-risk group; green: very low-risk group.*

*
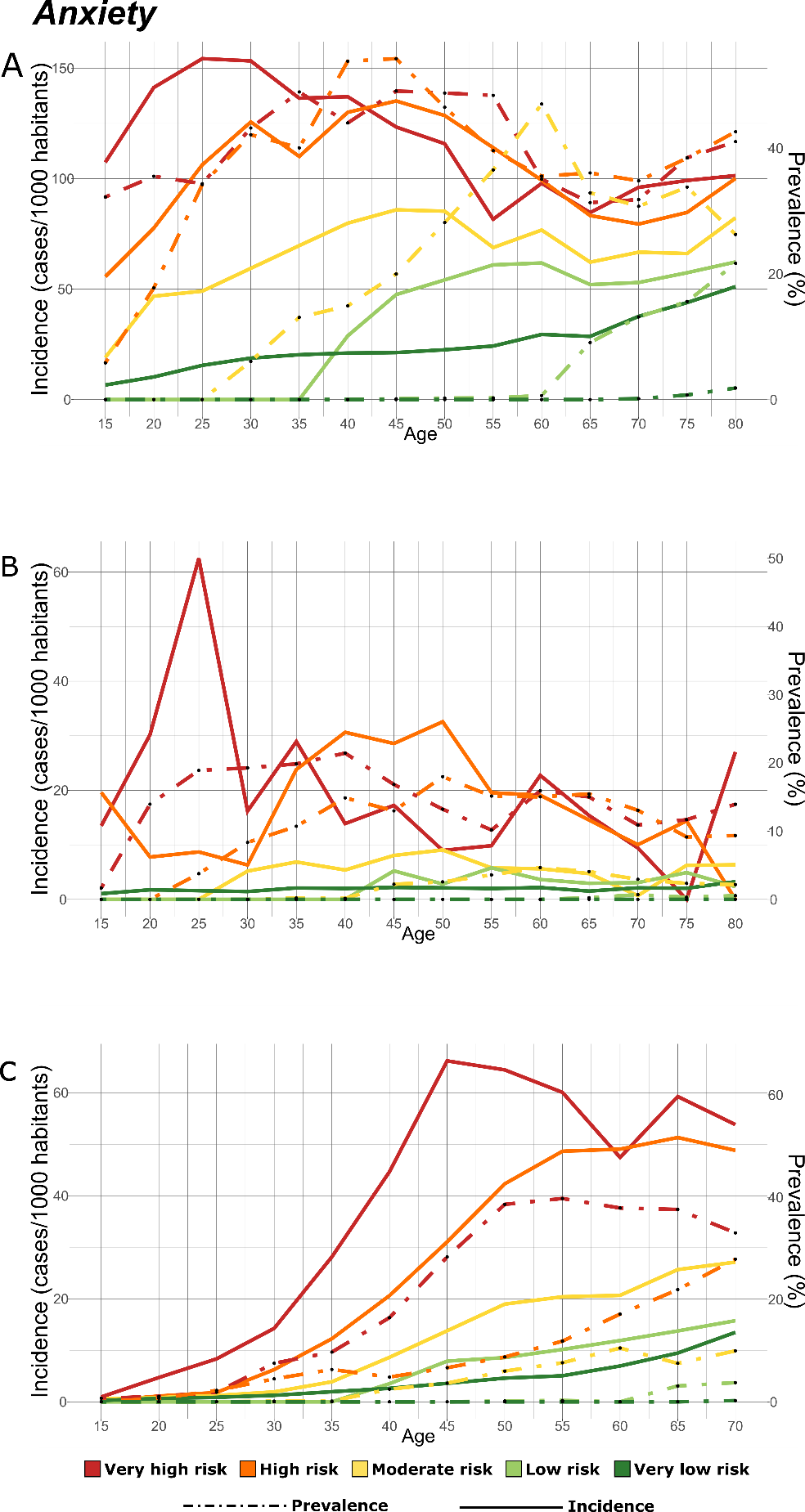
*

Figure S4 - Longitudinal analysis of disease prevalence and incidence of new onsets of anxiety related disorders (ICD-10-CM: F40-41). *Panel A) CHSS cohort; Panel B) THL cohort; Panel C)UKB cohort. Disease incidence is assessed in a 5-years interval, plotted in the left y-axis and represented with solid lines. Disease prevalence is plotted in the right y-axis and represented with dashed lines. The line colours correspond to the MADS risk pyramid tiers: red: very high-risk group; orange: high-risk group; yellow: medium-risk group; light green: low-risk group; green: very low-risk group.*

*
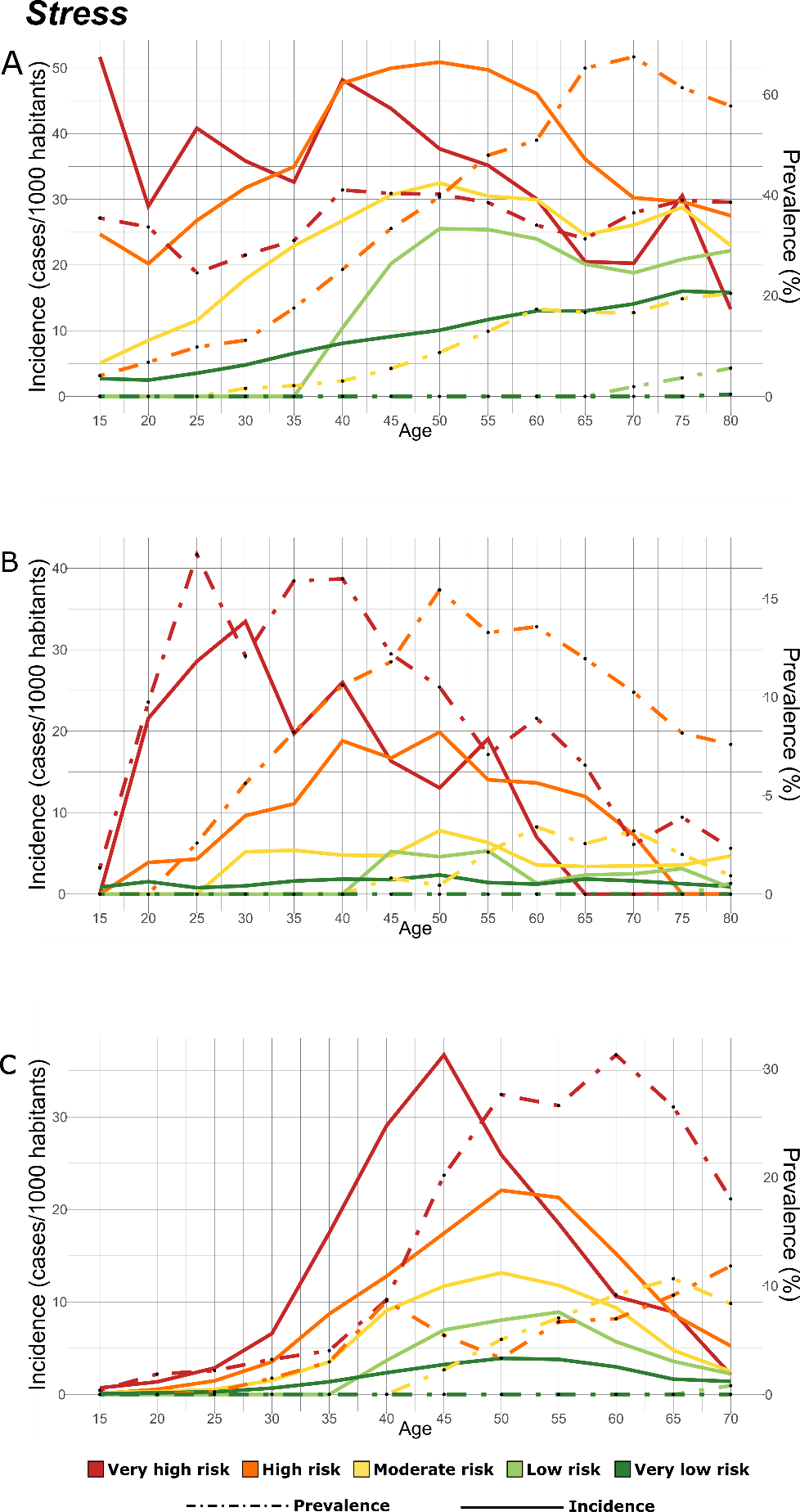
*

Figure S5 - Longitudinal analysis of disease prevalence and incidence of new onsets of stress related disorders (ICD-10-CM: F43). *Panel A) CHSS cohort; Panel B) THL cohort; Panel C)UKB cohort. Disease incidence is assessed in a 5-years interval, plotted in the left y-axis and represented with solid lines. Disease prevalence is plotted in the right y-axis and represented with dashed lines. The line colours correspond to the MADS risk pyramid tiers: red: very high-risk group; orange: high-risk group; yellow: medium-risk group; light green: low-risk group; green: very low-risk group.*

*
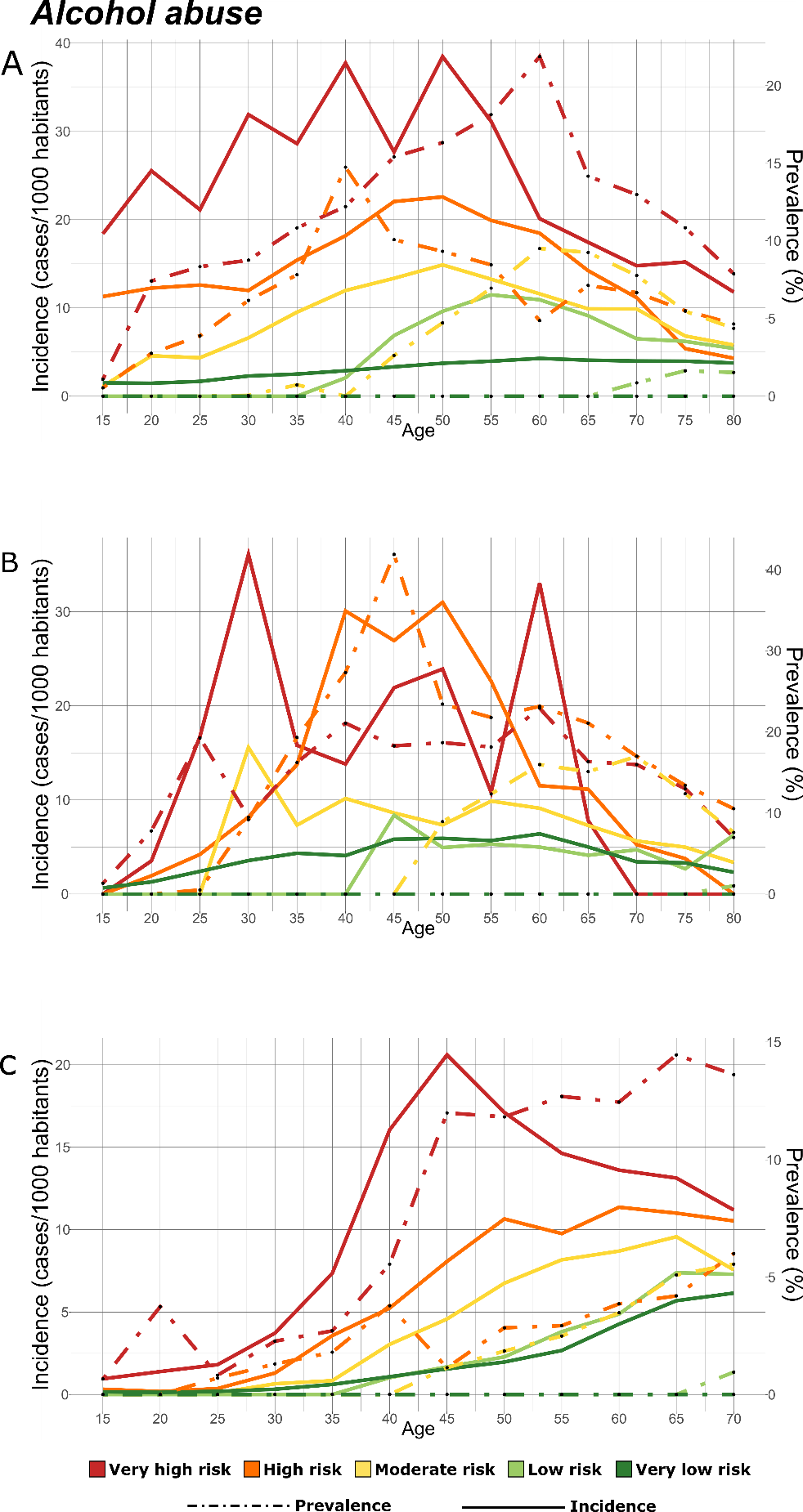
*

Figure S6 - Longitudinal analysis of disease prevalence and incidence of new onsets of mental disorders related to alcohol abuse (ICD-10-CM: F10). *Panel A) CHSS cohort; Panel B) THL cohort; Panel C)UKB cohort. Disease incidence is assessed in a 5-years interval, plotted in the left y-axis and represented with solid lines. Disease prevalence is plotted in the right y-axis and represented with dashed lines. The line colours correspond to the MADS risk pyramid tiers: red: very high-risk group; orange: high-risk group; yellow: medium-risk group; light green: low-risk group; green: very low-risk group.*

*
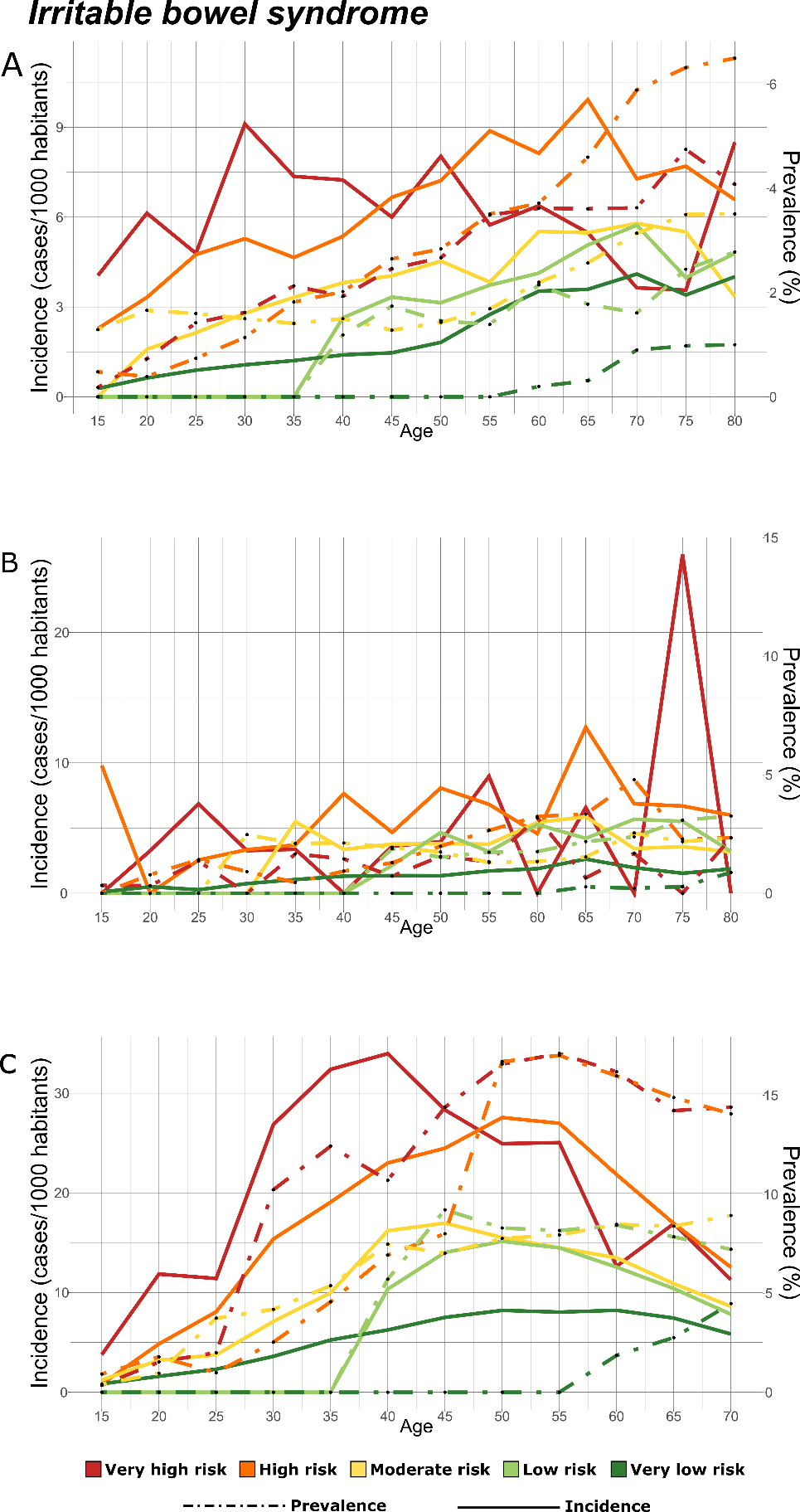
*

Figure S7 - Longitudinal analysis of disease prevalence and incidence of new onsets of irritable bowel syndrome (ICD-10-CM: K58). *Panel A) CHSS cohort; Panel B) THL cohort; Panel C)UKB cohort. Disease incidence is assessed in a 5-years interval, plotted in the left y-axis and represented with solid lines. Disease prevalence is plotted in the right y-axis and represented with dashed lines. The line colours correspond to the MADS risk pyramid tiers: red: very high-risk group; orange: high-risk group; yellow: medium-risk group; light green: low-risk group; green: very low-risk group.*

*
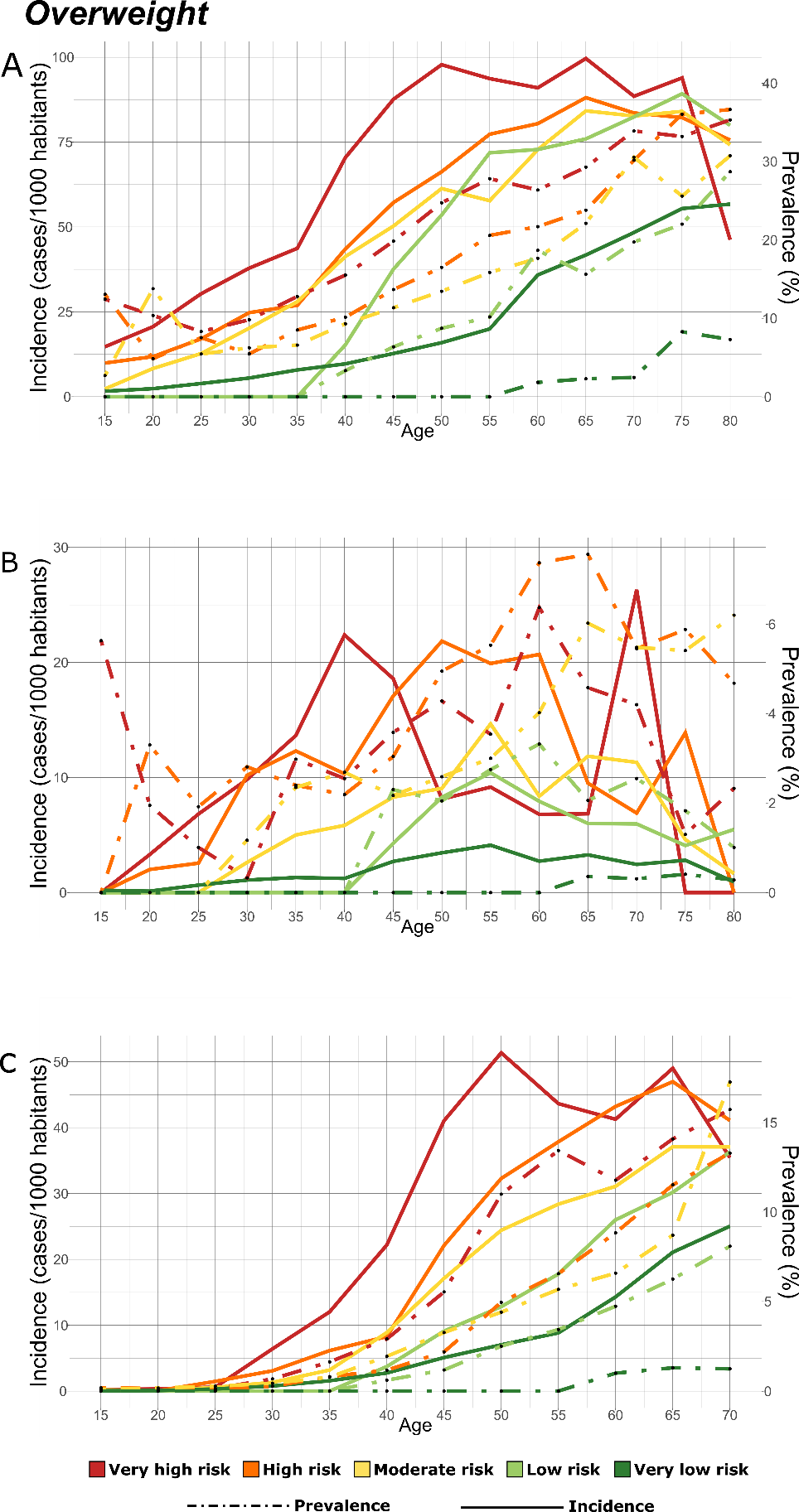
*

Figure S8 - Longitudinal analysis of disease prevalence and incidence of new onsets of overweight and obesity MDD (ICD-10-CM: E66). *Panel A) CHSS cohort; Panel B) THL cohort; Panel C)UKB cohort. Disease incidence is assessed in a 5-years interval, plotted in the left y-axis and represented with solid lines. Disease prevalence is plotted in the right y-axis and represented with dashed lines. The line colours correspond to the MADS risk pyramid tiers: red: very high-risk group; orange: high-risk group; yellow: medium-risk group; light green: low-risk group; green: very low-risk group.*

*
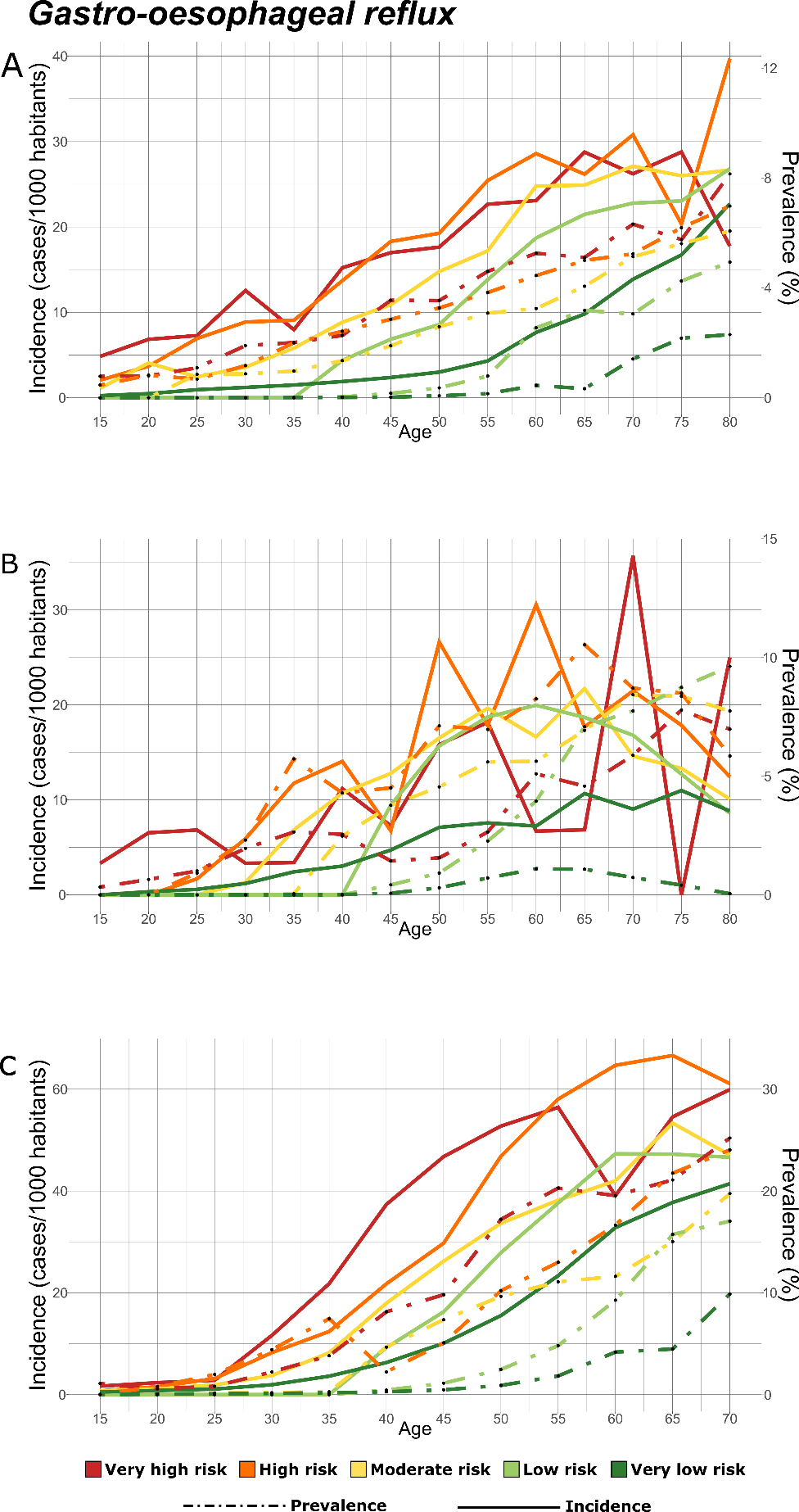
*

Figure S9 - Longitudinal analysis of disease prevalence and incidence of new onsets of gastro-oesophageal reflux (ICD-10-CM: K21). *Panel A) CHSS cohort; Panel B) THL cohort; Panel C)UKB cohort. Disease incidence is assessed in a 5-years interval, plotted in the left y-axis and represented with solid lines. Disease prevalence is plotted in the right y-axis and represented with dashed lines. The line colours correspond to the MADS risk pyramid tiers: red: very high-risk group; orange: high-risk group; yellow: medium-risk group; light green: low-risk group; green: very low-risk group.*

# Annex 3: Adjusted Disability Weights

### Table S28 – Disability weights associated to 88 ICD-10-CM diagnostic codes.

A/C indicates weather a disease is considered acute or chronic. DW stands for disability weight.

| A/C | DW Acute | DW Chronic | ICD10 | Disease Name |
| --- | --- | --- | --- | --- |
| A | 0.0497 | - | D64 | Other anemias |
| C | - | 0.0190 | E03 | Other hypothyroidism |
| C | - | 0.0388 | E66 | Overweight and obesity |
| C | - | 0.0388 | E78 | Disorders of lipoprotein metabolism and other lipidemias |
| C | - | 0.1071 | F10 | Alcohol related disorders |
| C | - | 0.1414 | F17 | Nicotine dependence |
| A/C | 0.7780 | 0.5880 | F20 | Schizophrenia |
| A/C | 0.4920 | 0.0320 | F31 | Bipolar disorder |
| C | - | 0.2187 | F32 | Major depressive disorder, single episode |
| C | - | 0.2187 | F33 | Major depressive disorder, recurrent |
| C | - | 0.1036 | F40 | Phobic anxiety disorders |
| C | - | 0.1036 | F41 | Other anxiety disorders |
| C | - | 0.1036 | F43 | Reaction to severe stress, and adjustment disorders |
| C | - | 0.1036 | F45 | Somatoform disorders |
| C | - | 0.4942 | G40 | Epilepsy and recurrent seizures |
| C | - | 0.4410 | G43 | Migraine |
| A | 0.0100 | - | G47 | Sleep disorders |
| C | - | 0.0100 | G56 | Mononeuropathies of upper limb |
| A | 0.0702 | - | H10 | Conjunctivitis |
| A | 0.0702 | - | H53 | Visual disturbances |
| A/C | 0.0130 | 0.0032 | H65 | Nonsuppurative otitis media |
| A/C | 0.0130 | 0.0032 | H66 | Suppurative and unspecified otitis media |
| A | 0.1130 | - | H81 | Disorders of vestibular function |
| C | - | 0.1492 | H91 | Other and unspecified hearing loss |
| A | 0.1492 | - | H93 | Other disorders of ear, not elsewhere classified |
| C | - | 0.0871 | I10 | Essential (primary) hypertension |
| C | - | 0.0871 | I49 | Other cardiac arrhythmias |
| C | - | 0.4320 | I63 | Cerebral infarction |
| C | - | 0.4320 | I69 | Sequelae of cerebrovascular disease |
| A | 0.0871 | - | I83 | Varicose veins of lower extremities |
| A | 0.0871 | - | I95 | Hypotension |
| A | 0.0258 | - | J00 | Acute nasopharyngitis [common cold] |
| A | 0.0258 | - | J01 | Acute sinusitis |
| A | 0.0258 | - | J03 | Acute tonsillitis |
| A | 0.0258 | - | J06 | Acute upper respiratory infections of multiple and unspecified sites |
| A | 0.0633 | - | J18 | Pneumonia, unspecified organism |
| A | 0.0633 | - | J20 | Acute bronchitis |
| C | - | 0.0808 | J30 | Vasomotor and allergic rhinitis |
| C | - | 0.0808 | J31 | Chronic rhinitis, nasopharyngitis and pharyngitis |
| C | - | 0.2673 | J44 | Other chronic obstructive pulmonary disease |
| C | - | 0.0414 | J45 | Asthma |
| C | - | 0.0298 | K21 | Gastro-esophageal reflux disease |
| A | 0.3240 | - | K29 | Gastritis and duodenitis |
| A | 0.3240 | - | K35 | Acute appendicitis |
| A | 0.3240 | - | K43 | Ventral hernia |
| A | 0.3240 | - | K44 | Diaphragmatic hernia |
| A | 0.2310 | - | K52 | Other and unspecified noninfective gastroenteritis and colitis |
| A | 0.3240 | - | K56 | Paralytic ileus and intestinal obstruction without hernia |
| C | - | 0.0100 | K58 | Irritable bowel syndrome |
| A | 0.0100 | - | K59 | Other functional intestinal disorders |
| A | 0.5010 | - | K60 | Fissure and fistula of anal and rectal regions |
| A | 0.0100 | - | K62 | Other diseases of anus and rectum |
| C | - | 0.1780 | K76 | Other diseases of liver |
| A | 0.3240 | - | K80 | Cholelithiasis |
| C | - | 0.2637 | L20 | Atopic dermatitis |
| A | 0.0110 | - | L29 | Pruritus |
| A | 0.0110 | - | L30 | Other and unspecified dermatitis |
| C | - | 0.2637 | L40 | Psoriasis |
| A | 0.0914 | - | L50 | Urticaria |
| C | - | 0.0110 | L57 | Skin changes due to chronic exposure to nonionizing radiation |
| A | 0.0110 | - | L72 | Follicular cysts of skin and subcutaneous tissue |
| A | 0.0110 | - | L82 | Seborrheic keratosis |
| A | 0.0110 | - | L98 | Other disorders of skin and subcutaneous tissue, not elsewhere classified |
| C | - | 0.2472 | M06 | Other rheumatoid arthritis |
| C | - | 0.2950 | M10 | Gout |
| C | - | 0.0380 | M15 | Polyosteoarthritis |
| C | - | 0.0380 | M17 | Osteoarthritis of knee |
| C | - | 0.1700 | M20 | Acquired deformities of fingers and toes |
| C | - | 0.1700 | M24 | Other specific joint derangements |
| C | - | 0.1700 | M35 | Other systemic involvement of connective tissue |
| C | - | 0.1260 | M48 | Other spondylopathies |
| C | - | 0.1260 | M51 | Thoracic, thoracolumbar, and lumbosacral intervertebral disc disorders |
| A | 0.1064 | - | M54 | Dorsalgia |
| A | 0.1700 | - | M72 | Fibroblastic disorders |
| A | 0.1700 | - | M75 | Shoulder lesions |
| A | 0.1700 | - | M77 | Other enthesopathies |
| A | 0.1700 | - | M79 | Other and unspecified soft tissue disorders, not elsewhere classified |
| A | 0.0775 | - | N17 | Acute kidney failure |
| C | - | 0.3375 | N18 | Chronic kidney disease (CKD) |
| A | 0.0347 | - | N30 | Cystitis |
| C | - | 0.0670 | N40 | Benign prostatic hyperplasia |
| A | 0.0110 | - | N64 | Other disorders of breast |
| A | 0.0110 | - | N76 | Other inflammation of vagina and vulva |
| C | - | 0.0308 | N81 | Female genital prolapse |
| A | 0.0110 | - | N84 | Polyp of female genital tract |
| C | - | 0.0110 | N92 | Excessive, frequent and irregular menstruation |
| A | 0.0110 | - | N94 | Pain and other conditions associated with female genital organs and menstrual cycle |
| C | - | 0.0110 | N95 | Menopausal and other perimenopausal disorders |

# Annex 4: Probabilities of Relevance

### Table S29 – Probabilities of relevance associated to 88 ICD-10-CM diagnostic codes.

Age interval indicates the patient’s age. Disease onset interval indicates the patients age at disease onset. Interval 1: 0-20 years; Interval 2: 20-40 years; Interval 3: 40-60 years; Interval 4: >60 years. PR stands for probability of relevance.

| Disease | Age Interval | Disease Onset Interval | PR |
| --- | --- | --- | --- |
| D64 | 1 | 1 | 0.207629 |
| E03 | 1 | 1 | 0.490952 |
| E66 | 1 | 1 | 0.514122 |
| E78 | 1 | 1 | 0.242673 |
| F10 | 1 | 1 | 0.537501 |
| F17 | 1 | 1 | 0.538888 |
| F41 | 1 | 1 | 1 |
| F43 | 1 | 1 | 0.996442 |
| G40 | 1 | 1 | 0.357502 |
| G43 | 1 | 1 | 0.587172 |
| G56 | 1 | 1 | 0.241626 |
| H10 | 1 | 1 | 0.281522 |
| H53 | 1 | 1 | 0.538474 |
| H65 | 1 | 1 | 0.537491 |
| H66 | 1 | 1 | 0.487149 |
| H81 | 1 | 1 | 0.217475 |
| H91 | 1 | 1 | 0.168141 |
| H93 | 1 | 1 | 0.158599 |
| I10 | 1 | 1 | 0.211192 |
| I49 | 1 | 1 | 0.222648 |
| I63 | 1 | 1 | 0.260996 |
| I83 | 1 | 1 | 0.223732 |
| I95 | 1 | 1 | 0.306704 |
| J00 | 1 | 1 | 0.257687 |
| J01 | 1 | 1 | 0.546747 |
| J03 | 1 | 1 | 0.72973 |
| J06 | 1 | 1 | 0.584992 |
| J18 | 1 | 1 | 0.482417 |
| J20 | 1 | 1 | 0.471248 |
| J30 | 1 | 1 | 0.703677 |
| J44 | 1 | 1 | 0.317264 |
| J45 | 1 | 1 | 0.750012 |
| K21 | 1 | 1 | 0.426771 |
| K29 | 1 | 1 | 0.21998 |
| K43 | 1 | 1 | 0.460557 |
| K44 | 1 | 1 | 0.244155 |
| K52 | 1 | 1 | 0.22367 |
| K56 | 1 | 1 | 0.179363 |
| K58 | 1 | 1 | 0.409958 |
| K59 | 1 | 1 | 0.23038 |
| K60 | 1 | 1 | 0.181343 |
| K62 | 1 | 1 | 0.232775 |
| K76 | 1 | 1 | 0.301806 |
| K80 | 1 | 1 | 0.236866 |
| L20 | 1 | 1 | 0.185839 |
| L29 | 1 | 1 | 0.278717 |
| L30 | 1 | 1 | 0.548071 |
| L40 | 1 | 1 | 0.096611 |
| L50 | 1 | 1 | 0.339331 |
| L57 | 1 | 1 | 0.275775 |
| L72 | 1 | 1 | 0.22363 |
| L82 | 1 | 1 | 0.289739 |
| L98 | 1 | 1 | 0.193764 |
| M10 | 1 | 1 | 0.533441 |
| M15 | 1 | 1 | 0.380628 |
| M17 | 1 | 1 | 0.223994 |
| M20 | 1 | 1 | 0.242078 |
| M48 | 1 | 1 | 0.185253 |
| M51 | 1 | 1 | 0.153944 |
| M54 | 1 | 1 | 0.358052 |
| M72 | 1 | 1 | 0.216581 |
| M75 | 1 | 1 | 0.382661 |
| M77 | 1 | 1 | 0.328523 |
| M79 | 1 | 1 | 0.676191 |
| N17 | 1 | 1 | 0.299834 |
| N18 | 1 | 1 | 0.250667 |
| N30 | 1 | 1 | 0.427956 |
| N40 | 1 | 1 | 0.27723 |
| N64 | 1 | 1 | 0.61271 |
| N76 | 1 | 1 | 0.233089 |
| N92 | 1 | 1 | 0.198923 |
| N94 | 1 | 1 | 0.504627 |
| N95 | 1 | 1 | 0.314981 |
| F20 | 1 | 1 | 1 |
| F31 | 1 | 1 | 0.617285 |
| F40 | 1 | 1 | 0.996332 |
| F45 | 1 | 1 | 0.191534 |
| G47 | 1 | 1 | 0.431321 |
| I69 | 1 | 1 | 0.105805 |
| J31 | 1 | 1 | 0.482109 |
| K35 | 1 | 1 | 0.128864 |
| M06 | 1 | 1 | 0.15675 |
| M24 | 1 | 1 | 0.268155 |
| M35 | 1 | 1 | 0.361547 |
| N81 | 1 | 1 | 0.33795 |
| N84 | 1 | 1 | 0.273644 |
| D64 | 2 | 2 | 0.145307 |
| E03 | 2 | 2 | 0.666467 |
| E66 | 2 | 2 | 0.90324 |
| E78 | 2 | 2 | 0.607976 |
| F10 | 2 | 2 | 0.932132 |
| F17 | 2 | 2 | 0.679072 |
| F41 | 2 | 2 | 0.666667 |
| F43 | 2 | 2 | 1 |
| G40 | 2 | 2 | 0.069491 |
| G43 | 2 | 2 | 0.522212 |
| G56 | 2 | 2 | 0.002524 |
| H10 | 2 | 2 | 0.177709 |
| H53 | 2 | 2 | 0.369519 |
| H65 | 2 | 2 | 0.088946 |
| H66 | 2 | 2 | 0.333856 |
| H81 | 2 | 2 | 0.001532 |
| H91 | 2 | 2 | 0.273224 |
| H93 | 2 | 2 | 0.08316 |
| I10 | 2 | 2 | 0.313948 |
| I49 | 2 | 2 | 0.011699 |
| I63 | 2 | 2 | 0.025087 |
| I83 | 2 | 2 | 0.004941 |
| I95 | 2 | 2 | 0.36688 |
| J00 | 2 | 2 | 0.255248 |
| J01 | 2 | 2 | 0.593139 |
| J03 | 2 | 2 | 0.30154 |
| J06 | 2 | 2 | 0.750867 |
| J18 | 2 | 2 | 0.359907 |
| J20 | 2 | 2 | 0.337761 |
| J30 | 2 | 2 | 0.664874 |
| J44 | 2 | 2 | 0.016355 |
| J45 | 2 | 2 | 0.58681 |
| K21 | 2 | 2 | 0.725603 |
| K29 | 2 | 2 | 0.216038 |
| K43 | 2 | 2 | 0.006751 |
| K44 | 2 | 2 | 0.007032 |
| K52 | 2 | 2 | 0.094769 |
| K56 | 2 | 2 | 0.037373 |
| K58 | 2 | 2 | 0.339967 |
| K59 | 2 | 2 | 0.019506 |
| K60 | 2 | 2 | 0.269061 |
| K62 | 2 | 2 | 0.118576 |
| K76 | 2 | 2 | 0.008777 |
| K80 | 2 | 2 | 0.00885 |
| L20 | 2 | 2 | 0.029375 |
| L29 | 2 | 2 | 0.077698 |
| L30 | 2 | 2 | 0.321978 |
| L40 | 2 | 2 | 0.36384 |
| L50 | 2 | 2 | 0.004706 |
| L57 | 2 | 2 | 0.014397 |
| L72 | 2 | 2 | 0.002464 |
| L82 | 2 | 2 | 0.025031 |
| L98 | 2 | 2 | 0.004672 |
| M10 | 2 | 2 | 0.229375 |
| M15 | 2 | 2 | 0.021844 |
| M17 | 2 | 2 | 0.008602 |
| M20 | 2 | 2 | 0.025575 |
| M48 | 2 | 2 | 0.543157 |
| M51 | 2 | 2 | 0.669599 |
| M54 | 2 | 2 | 1 |
| M72 | 2 | 2 | 0.005597 |
| M75 | 2 | 2 | 0.266992 |
| M77 | 2 | 2 | 0.268155 |
| M79 | 2 | 2 | 0.950369 |
| N17 | 2 | 2 | 0.126441 |
| N18 | 2 | 2 | 0.229945 |
| N30 | 2 | 2 | 0.13409 |
| N40 | 2 | 2 | 0.021651 |
| N64 | 2 | 2 | 0.036268 |
| N76 | 2 | 2 | 0.321532 |
| N92 | 2 | 2 | 0.611377 |
| N94 | 2 | 2 | 0.40996 |
| N95 | 2 | 2 | 0.020229 |
| F20 | 2 | 2 | 1 |
| F31 | 2 | 2 | 1 |
| F40 | 2 | 2 | 1 |
| F45 | 2 | 2 | 0.070387 |
| G47 | 2 | 2 | 0.664282 |
| I69 | 2 | 2 | 0.023645 |
| J31 | 2 | 2 | 0.027975 |
| K35 | 2 | 2 | 0.021178 |
| M06 | 2 | 2 | 0.182826 |
| M24 | 2 | 2 | 0.267584 |
| M35 | 2 | 2 | 0.26637 |
| N81 | 2 | 2 | 0.00845 |
| N84 | 2 | 2 | 0.026969 |
| D64 | 2 | 1 | 0.020895 |
| E03 | 2 | 1 | 0.639846 |
| E66 | 2 | 1 | 0.197175 |
| E78 | 2 | 1 | 0.5 |
| F10 | 2 | 1 | 0.50732 |
| F17 | 2 | 1 | 0.406307 |
| F41 | 2 | 1 | 0.699404 |
| F43 | 2 | 1 | 0.902009 |
| G40 | 2 | 1 | 0.071184 |
| G43 | 2 | 1 | 0.330547 |
| G56 | 2 | 1 | 0.059231 |
| H10 | 2 | 1 | 0.228742 |
| H53 | 2 | 1 | 0.113624 |
| H65 | 2 | 1 | 0.029636 |
| H66 | 2 | 1 | 0.003778 |
| H81 | 2 | 1 | 0.027335 |
| H91 | 2 | 1 | 0.018025 |
| H93 | 2 | 1 | 0.00194 |
| I10 | 2 | 1 | 0.012804 |
| I49 | 2 | 1 | 0.014845 |
| I63 | 2 | 1 | 0.054555 |
| I83 | 2 | 1 | 0.02105 |
| I95 | 2 | 1 | 0.12384 |
| J00 | 2 | 1 | 0.118451 |
| J01 | 2 | 1 | 0.009064 |
| J03 | 2 | 1 | 0.037438 |
| J06 | 2 | 1 | 0.196419 |
| J18 | 2 | 1 | 0.174266 |
| J20 | 2 | 1 | 0.081901 |
| J30 | 2 | 1 | 0.036414 |
| J44 | 2 | 1 | 0.017101 |
| J45 | 2 | 1 | 0.407349 |
| K21 | 2 | 1 | 0.400123 |
| K29 | 2 | 1 | 0.037 |
| K43 | 2 | 1 | 0.021352 |
| K44 | 2 | 1 | 0.002154 |
| K52 | 2 | 1 | 0.035781 |
| K56 | 2 | 1 | 0.058305 |
| K58 | 2 | 1 | 0.143014 |
| K59 | 2 | 1 | 0.025896 |
| K60 | 2 | 1 | 0.096265 |
| K62 | 2 | 1 | 0.166295 |
| K76 | 2 | 1 | 0.012471 |
| K80 | 2 | 1 | 0.02605 |
| L20 | 2 | 1 | 0.04015 |
| L29 | 2 | 1 | 0.029133 |
| L30 | 2 | 1 | 0.058341 |
| L40 | 2 | 1 | 0.099776 |
| L50 | 2 | 1 | 0.012935 |
| L57 | 2 | 1 | 0.010156 |
| L72 | 2 | 1 | 0.004805 |
| L82 | 2 | 1 | 0.010051 |
| L98 | 2 | 1 | 0.018125 |
| M10 | 2 | 1 | 0.039846 |
| M15 | 2 | 1 | 0.021502 |
| M17 | 2 | 1 | 0.05786 |
| M20 | 2 | 1 | 0.01368 |
| M48 | 2 | 1 | 0.557425 |
| M51 | 2 | 1 | 0.417676 |
| M54 | 2 | 1 | 0.370174 |
| M72 | 2 | 1 | 0.019751 |
| M75 | 2 | 1 | 0.006166 |
| M77 | 2 | 1 | 0.00928 |
| M79 | 2 | 1 | 0.210014 |
| N17 | 2 | 1 | 0.012911 |
| N18 | 2 | 1 | 0.327588 |
| N30 | 2 | 1 | 0.088923 |
| N40 | 2 | 1 | 0.238988 |
| N64 | 2 | 1 | 0.02793 |
| N76 | 2 | 1 | 0.310994 |
| N92 | 2 | 1 | 0.080761 |
| N94 | 2 | 1 | 0.181871 |
| N95 | 2 | 1 | 0.006917 |
| F20 | 2 | 1 | 0.928062 |
| F31 | 2 | 1 | 0.666667 |
| F40 | 2 | 1 | 0.666667 |
| F45 | 2 | 1 | 0.028975 |
| I69 | 2 | 1 | 8.48E-05 |
| J31 | 2 | 1 | 0.036557 |
| K35 | 2 | 1 | 0.019644 |
| M06 | 2 | 1 | 0.017095 |
| M24 | 2 | 1 | 0.010196 |
| M35 | 2 | 1 | 0.287653 |
| N81 | 2 | 1 | 0.012432 |
| N84 | 2 | 1 | 0.005068 |
| D64 | 3 | 3 | 0.000233 |
| E03 | 3 | 3 | 0.581504 |
| E66 | 3 | 3 | 0.215081 |
| E78 | 3 | 3 | 0.641215 |
| F10 | 3 | 3 | 0.655622 |
| F17 | 3 | 3 | 0.337533 |
| F41 | 3 | 3 | 1 |
| F43 | 3 | 3 | 1 |
| G40 | 3 | 3 | 7.64E-05 |
| G43 | 3 | 3 | 0.127064 |
| G56 | 3 | 3 | 0.02573 |
| H10 | 3 | 3 | 0.033825 |
| H53 | 3 | 3 | 0.003988 |
| H65 | 3 | 3 | 0.002786 |
| H66 | 3 | 3 | 0.336791 |
| H81 | 3 | 3 | 0.049376 |
| H91 | 3 | 3 | 0.006947 |
| H93 | 3 | 3 | 0.046048 |
| I10 | 3 | 3 | 0.666667 |
| I49 | 3 | 3 | 0.258034 |
| I63 | 3 | 3 | 0.000847 |
| I83 | 3 | 3 | 0.154023 |
| I95 | 3 | 3 | 0.006157 |
| J00 | 3 | 3 | 0.335306 |
| J01 | 3 | 3 | 0.153879 |
| J03 | 3 | 3 | 0.001042 |
| J06 | 3 | 3 | 0.333746 |
| J18 | 3 | 3 | 0.00034 |
| J20 | 3 | 3 | 0.048305 |
| J30 | 3 | 3 | 0.067269 |
| J44 | 3 | 3 | 0.200251 |
| J45 | 3 | 3 | 0.134008 |
| K21 | 3 | 3 | 0.804697 |
| K29 | 3 | 3 | 0.489027 |
| K43 | 3 | 3 | 0.000771 |
| K44 | 3 | 3 | 0.133885 |
| K52 | 3 | 3 | 0.437633 |
| K56 | 3 | 3 | 0.023764 |
| K58 | 3 | 3 | 0.838628 |
| K59 | 3 | 3 | 0.466593 |
| K60 | 3 | 3 | 0.00051 |
| K62 | 3 | 3 | 0.131354 |
| K76 | 3 | 3 | 0.003054 |
| K80 | 3 | 3 | 6.96E-05 |
| L20 | 3 | 3 | 0.014986 |
| L29 | 3 | 3 | 0.244383 |
| L30 | 3 | 3 | 0.079878 |
| L40 | 3 | 3 | 0.001048 |
| L50 | 3 | 3 | 0.000772 |
| L57 | 3 | 3 | 0.002291 |
| L72 | 3 | 3 | 0.002304 |
| L82 | 3 | 3 | 0.014331 |
| L98 | 3 | 3 | 0.043954 |
| M10 | 3 | 3 | 0.001691 |
| M15 | 3 | 3 | 0.01642 |
| M17 | 3 | 3 | 3.03E-05 |
| M20 | 3 | 3 | 0.001621 |
| M48 | 3 | 3 | 0.088179 |
| M51 | 3 | 3 | 0.139584 |
| M54 | 3 | 3 | 0.953653 |
| M72 | 3 | 3 | 0.002093 |
| M75 | 3 | 3 | 0.580773 |
| M77 | 3 | 3 | 0.100945 |
| M79 | 3 | 3 | 0.784598 |
| N17 | 3 | 3 | 0.027212 |
| N18 | 3 | 3 | 0.062883 |
| N30 | 3 | 3 | 0.327674 |
| N40 | 3 | 3 | 0.349648 |
| N64 | 3 | 3 | 0.0568 |
| N76 | 3 | 3 | 0.004811 |
| N92 | 3 | 3 | 0.03493 |
| N94 | 3 | 3 | 0.001314 |
| N95 | 3 | 3 | 0.279447 |
| F20 | 3 | 3 | 0.666667 |
| F31 | 3 | 3 | 1 |
| F40 | 3 | 3 | 0.336234 |
| F45 | 3 | 3 | 0.632903 |
| G47 | 3 | 3 | 0.666667 |
| I69 | 3 | 3 | 0.029474 |
| J31 | 3 | 3 | 0.001198 |
| K35 | 3 | 3 | 0.000174 |
| M06 | 3 | 3 | 0.002286 |
| M24 | 3 | 3 | 0.002296 |
| M35 | 3 | 3 | 0.334373 |
| N81 | 3 | 3 | 0.005425 |
| N84 | 3 | 3 | 0.001203 |
| D64 | 3 | 2 | 0.009784 |
| E03 | 3 | 2 | 0.581504 |
| E66 | 3 | 2 | 0.083618 |
| E78 | 3 | 2 | 0.210063 |
| F10 | 3 | 2 | 0.633333 |
| F17 | 3 | 2 | 0.099121 |
| F41 | 3 | 2 | 0.56674 |
| F43 | 3 | 2 | 0.600073 |
| G40 | 3 | 2 | 0.000487 |
| G43 | 3 | 2 | 0.133108 |
| G56 | 3 | 2 | 0.002473 |
| H10 | 3 | 2 | 0.011445 |
| H53 | 3 | 2 | 0.01428 |
| H65 | 3 | 2 | 0.016838 |
| H66 | 3 | 2 | 0.005995 |
| H81 | 3 | 2 | 0.010243 |
| H91 | 3 | 2 | 0.136588 |
| H93 | 3 | 2 | 4.27E-05 |
| I10 | 3 | 2 | 0.101343 |
| I49 | 3 | 2 | 0.011096 |
| I63 | 3 | 2 | 0.01957 |
| I83 | 3 | 2 | 0.005257 |
| I95 | 3 | 2 | 0.015927 |
| J00 | 3 | 2 | 0.002233 |
| J01 | 3 | 2 | 0.03338 |
| J03 | 3 | 2 | 0.004185 |
| J06 | 3 | 2 | 0.054189 |
| J18 | 3 | 2 | 0.004189 |
| J20 | 3 | 2 | 0.014529 |
| J30 | 3 | 2 | 0.002377 |
| J44 | 3 | 2 | 0.149343 |
| J45 | 3 | 2 | 0.234611 |
| K21 | 3 | 2 | 0.44033 |
| K29 | 3 | 2 | 0.070094 |
| K43 | 3 | 2 | 0.00893 |
| K44 | 3 | 2 | 0.114697 |
| K52 | 3 | 2 | 0.12342 |
| K56 | 3 | 2 | 0.006584 |
| K58 | 3 | 2 | 0.142484 |
| K59 | 3 | 2 | 0.466605 |
| K60 | 3 | 2 | 0.011556 |
| K62 | 3 | 2 | 0.041986 |
| K76 | 3 | 2 | 0.009094 |
| K80 | 3 | 2 | 0.000283 |
| L20 | 3 | 2 | 0.006752 |
| L29 | 3 | 2 | 0.024105 |
| L30 | 3 | 2 | 0.040511 |
| L40 | 3 | 2 | 0.013543 |
| L50 | 3 | 2 | 0.00626 |
| L57 | 3 | 2 | 0.000322 |
| L72 | 3 | 2 | 0.012774 |
| L82 | 3 | 2 | 0.000152 |
| L98 | 3 | 2 | 0.010782 |
| M10 | 3 | 2 | 0.025132 |
| M15 | 3 | 2 | 0.03191 |
| M17 | 3 | 2 | 0.010026 |
| M20 | 3 | 2 | 0.026688 |
| M48 | 3 | 2 | 0.125639 |
| M51 | 3 | 2 | 0.079216 |
| M54 | 3 | 2 | 0.066833 |
| M72 | 3 | 2 | 0.008739 |
| M75 | 3 | 2 | 0.02658 |
| M77 | 3 | 2 | 0.020682 |
| M79 | 3 | 2 | 0.185461 |
| N17 | 3 | 2 | 0.030636 |
| N18 | 3 | 2 | 0.032062 |
| N30 | 3 | 2 | 0.0101 |
| N40 | 3 | 2 | 0.336266 |
| N64 | 3 | 2 | 0.041827 |
| N76 | 3 | 2 | 0.006632 |
| N92 | 3 | 2 | 0.034444 |
| N94 | 3 | 2 | 0.035663 |
| N95 | 3 | 2 | 0.127145 |
| F20 | 3 | 2 | 0.666667 |
| F31 | 3 | 2 | 0.666667 |
| F40 | 3 | 2 | 0.335923 |
| F45 | 3 | 2 | 0.299765 |
| I69 | 3 | 2 | 0.020813 |
| J31 | 3 | 2 | 0.053806 |
| K35 | 3 | 2 | 0.00056 |
| M06 | 3 | 2 | 0.008837 |
| M24 | 3 | 2 | 0.005525 |
| M35 | 3 | 2 | 0.00814 |
| N81 | 3 | 2 | 0.005645 |
| N84 | 3 | 2 | 0.013678 |
| D64 | 4 | 4 | 0.001988 |
| E03 | 4 | 4 | 6.69E-05 |
| E66 | 4 | 4 | 0.60924 |
| E78 | 4 | 4 | 0.334674 |
| F10 | 4 | 4 | 0.732118 |
| F17 | 4 | 4 | 0.089398 |
| F41 | 4 | 4 | 0.672721 |
| F43 | 4 | 4 | 0.665326 |
| G40 | 4 | 4 | 0.002705 |
| G43 | 4 | 4 | 0.002275 |
| G56 | 4 | 4 | 0.009524 |
| H10 | 4 | 4 | 0.004784 |
| H53 | 4 | 4 | 0.002745 |
| H65 | 4 | 4 | 0.015268 |
| H66 | 4 | 4 | 0.009448 |
| H81 | 4 | 4 | 0.001592 |
| H91 | 4 | 4 | 0.004356 |
| H93 | 4 | 4 | 0.019601 |
| I10 | 4 | 4 | 0.6761 |
| I49 | 4 | 4 | 0.190506 |
| I63 | 4 | 4 | 0.334625 |
| I83 | 4 | 4 | 4.58E-05 |
| I95 | 4 | 4 | 0.003859 |
| J00 | 4 | 4 | 0.012023 |
| J01 | 4 | 4 | 0.001793 |
| J03 | 4 | 4 | 0.015985 |
| J06 | 4 | 4 | 0.482069 |
| J18 | 4 | 4 | 0.192174 |
| J20 | 4 | 4 | 0.002659 |
| J30 | 4 | 4 | 0.000795 |
| J44 | 4 | 4 | 0.074403 |
| J45 | 4 | 4 | 0.000279 |
| K21 | 4 | 4 | 0.100767 |
| K29 | 4 | 4 | 0.205315 |
| K43 | 4 | 4 | 0.004221 |
| K44 | 4 | 4 | 0.00207 |
| K52 | 4 | 4 | 0.001679 |
| K56 | 4 | 4 | 0.302046 |
| K58 | 4 | 4 | 0.108629 |
| K59 | 4 | 4 | 0.589787 |
| K60 | 4 | 4 | 0.00122 |
| K62 | 4 | 4 | 0.001992 |
| K76 | 4 | 4 | 0.292126 |
| K80 | 4 | 4 | 4.49E-06 |
| L20 | 4 | 4 | 0.000321 |
| L29 | 4 | 4 | 0.00341 |
| L30 | 4 | 4 | 0.002894 |
| L40 | 4 | 4 | 0.003798 |
| L50 | 4 | 4 | 0.001382 |
| L57 | 4 | 4 | 0.002819 |
| L72 | 4 | 4 | 0.005499 |
| L82 | 4 | 4 | 0.004651 |
| L98 | 4 | 4 | 0.001031 |
| M10 | 4 | 4 | 0.30812 |
| M15 | 4 | 4 | 0.357456 |
| M17 | 4 | 4 | 0.000121 |
| M20 | 4 | 4 | 0.001677 |
| M48 | 4 | 4 | 0.33424 |
| M51 | 4 | 4 | 0.024462 |
| M54 | 4 | 4 | 0.757775 |
| M72 | 4 | 4 | 0.003851 |
| M75 | 4 | 4 | 0.000228 |
| M77 | 4 | 4 | 0.001568 |
| M79 | 4 | 4 | 0.341395 |
| N17 | 4 | 4 | 0.074247 |
| N18 | 4 | 4 | 0.067345 |
| N30 | 4 | 4 | 0.334153 |
| N40 | 4 | 4 | 0.00023 |
| N64 | 4 | 4 | 0.011165 |
| N76 | 4 | 4 | 0.011783 |
| N92 | 4 | 4 | 0.006472 |
| N94 | 4 | 4 | 0.009572 |
| N95 | 4 | 4 | 0.100244 |
| F20 | 4 | 4 | 1 |
| F31 | 4 | 4 | 0.705556 |
| F40 | 4 | 4 | 0.018261 |
| F45 | 4 | 4 | 0.007279 |
| G47 | 4 | 4 | 0.334086 |
| I69 | 4 | 4 | 0.336072 |
| J31 | 4 | 4 | 0.007434 |
| K35 | 4 | 4 | 0.310043 |
| M06 | 4 | 4 | 0.001018 |
| M24 | 4 | 4 | 0.014846 |
| M35 | 4 | 4 | 0.008497 |
| N81 | 4 | 4 | 0.000114 |
| N84 | 4 | 4 | 0.001004 |
| D64 | 4 | 3 | 0.031453 |
| E03 | 4 | 3 | 1.28E-05 |
| E66 | 4 | 3 | 0.001795 |
| E78 | 4 | 3 | 0.002176 |
| F10 | 4 | 3 | 0.620467 |
| F17 | 4 | 3 | 0.066422 |
| F41 | 4 | 3 | 0.433664 |
| F43 | 4 | 3 | 0.843938 |
| G40 | 4 | 3 | 0.002973 |
| G43 | 4 | 3 | 0.007675 |
| G56 | 4 | 3 | 0.002237 |
| H10 | 4 | 3 | 0.030127 |
| H53 | 4 | 3 | 0.341754 |
| H65 | 4 | 3 | 0.023073 |
| H66 | 4 | 3 | 0.010569 |
| H81 | 4 | 3 | 0.00763 |
| H91 | 4 | 3 | 0.00385 |
| H93 | 4 | 3 | 0.0425 |
| I10 | 4 | 3 | 0.00061 |
| I49 | 4 | 3 | 0.006614 |
| I63 | 4 | 3 | 0.008991 |
| I83 | 4 | 3 | 0.000473 |
| I95 | 4 | 3 | 0.01024 |
| J00 | 4 | 3 | 0.018283 |
| J01 | 4 | 3 | 0.009354 |
| J03 | 4 | 3 | 0.037861 |
| J06 | 4 | 3 | 0.00273 |
| J18 | 4 | 3 | 0.008017 |
| J20 | 4 | 3 | 0.021369 |
| J30 | 4 | 3 | 0.003008 |
| J44 | 4 | 3 | 0.026563 |
| J45 | 4 | 3 | 0.001518 |
| K21 | 4 | 3 | 0.001271 |
| K29 | 4 | 3 | 0.002406 |
| K43 | 4 | 3 | 0.005458 |
| K44 | 4 | 3 | 0.029245 |
| K52 | 4 | 3 | 0.008246 |
| K56 | 4 | 3 | 0.01696 |
| K58 | 4 | 3 | 0.011087 |
| K59 | 4 | 3 | 0.075915 |
| K60 | 4 | 3 | 0.020382 |
| K62 | 4 | 3 | 0.002944 |
| K76 | 4 | 3 | 0.018396 |
| K80 | 4 | 3 | 0.00105 |
| L20 | 4 | 3 | 0.005096 |
| L29 | 4 | 3 | 0.007139 |
| L30 | 4 | 3 | 0.005837 |
| L40 | 4 | 3 | 0.005635 |
| L50 | 4 | 3 | 0.019405 |
| L57 | 4 | 3 | 0.015421 |
| L72 | 4 | 3 | 0.014275 |
| L82 | 4 | 3 | 0.011029 |
| L98 | 4 | 3 | 0.030898 |
| M10 | 4 | 3 | 0.010196 |
| M15 | 4 | 3 | 0.012109 |
| M17 | 4 | 3 | 0.006472 |
| M20 | 4 | 3 | 0.002886 |
| M48 | 4 | 3 | 0.336163 |
| M51 | 4 | 3 | 0.012603 |
| M54 | 4 | 3 | 0.231507 |
| M72 | 4 | 3 | 0.011313 |
| M75 | 4 | 3 | 0.204019 |
| M77 | 4 | 3 | 0.003613 |
| M79 | 4 | 3 | 0.026842 |
| N17 | 4 | 3 | 0.086403 |
| N18 | 4 | 3 | 0.031897 |
| N30 | 4 | 3 | 0.005342 |
| N40 | 4 | 3 | 0.001307 |
| N64 | 4 | 3 | 0.012489 |
| N76 | 4 | 3 | 0.025574 |
| N92 | 4 | 3 | 0.008984 |
| N94 | 4 | 3 | 0.009884 |
| N95 | 4 | 3 | 0.0008 |
| F20 | 4 | 3 | 0.770102 |
| F31 | 4 | 3 | 0.706754 |
| F40 | 4 | 3 | 0.036717 |
| F45 | 4 | 3 | 0.00867 |
| G47 | 4 | 3 | 0.284504 |
| I69 | 4 | 3 | 0.35072 |
| J31 | 4 | 3 | 0.011781 |
| K35 | 4 | 3 | 0.31619 |
| M06 | 4 | 3 | 0.007894 |
| M24 | 4 | 3 | 0.115725 |
| M35 | 4 | 3 | 0.018638 |
| N81 | 4 | 3 | 0.005471 |
| N84 | 4 | 3 | 0.002112 |

# Annex 5: MADS Pseudocode

*# For each patient in the database, calculate the MADS*

**for each patient in the database:**

**mads =** 1 *# Initialize the MADS for the patient*

*# For each disease the patient has, calculate its contribution to the score*

**for each disease in patient:**

**P** = 0 *# Initialize the probability of relevance for the disease*

**dw** = 0 *# Initialize the disability weight*

**dw_score** = 0 *# Initialize the score contribution from the disability weight*

*# Determine the probability of relevance based on the patient's age and the disease onset age*

**if patient_age <= 20:**

P = get_probability('relevance_interval_1', disease_name)

**elif patient_age <= 40:**

if disease_onset <= 40:

P = get_probability('relevance_interval_22', disease_name)

elif disease_onset <= 20:

P = get_probability('relevance_interval_21', disease_name)

**elif patient_age <= 60:**

if disease_onset <= 60:

P = get_probability('relevance_interval_33', disease_name)

elif disease_onset <= 40:

P = get_probability('relevance_interval_32', disease_name)

**elif patient_age > 60:**

if disease_onset > 60:

P = get_probability('relevance_interval_44', disease_name)

elif disease_onset <= 60:

P = get_probability('relevance_interval_43', disease_name)

*# Retrieve disability weight (DW) information for the current disease*

**DW** = get_disability_weight(disease_name)

*# Adjust the disability weight based on whether the condition is chronic or acute*

**if DW['A/C'] == 'C':**

dw = DW['Adjusted DW Chronic']

**elif DW['A/C'] == 'A': *#*** *DWs for the acute diseases diagnosed more than 12 months before the MADS assessment were arbitrary set to 0 (no disability)*

if patient_age - disease_onset <= 1:

dw = DW['Adjusted DW Acute']

else:

dw = 0

**elif DW['A/C'] == 'A/C':** *# For diseases with varying disability weights for acute and chronic stages acute disability weight is applied if the disease onset was within the past year before the MADS assessment, otherwise the chronic disability weight is used*

if patient_age - disease_onset <= 1:

dw = DW['Adjusted DW Acute']

else:

dw = DW['Adjusted DW Chronic']

*# Calculate the score contribution for this disease and update the mads*

dw_score = P * dw

mads = mads * (1 - dw_score)
