# Supplementary material for: Multicentric Assessment of a Multimorbidity-Adjusted Disability Score to Stratify Depression-Related Risks Using Temporal Disease Maps: Instrument Validation Study
Source: J Med Internet Res. 2024 Jun 24;26:e53162. doi: 10.2196/53162 (PMC11231623; doi:10.2196/53162)
Supplement: Multimedia Appendix 2 [file jmir_v26i1e53162_app2.docx]

| **Disease** | **Risk pyramid  tiers** | **CHSS** | | | | | | | | **THL** | | | | | | | | **UKB** | | | | | | | |
| --- | --- | --- | --- | --- | --- | --- | --- | --- | --- | --- | --- | --- | --- | --- | --- | --- | --- | --- | --- | --- | --- | --- | --- | --- | --- |
|  |  | **Prevalence (%)** | | | | **Incidence (‰)** | | | | **Prevalence (%)** | | | | **Incidence (‰)** | | | | **Prevalence (%)** | | | | **Incidence (‰)** | | | |
|  |  | **20** | **40** | **60** | **70** | **20** | **40** | **60** | **70** | **20** | **40** | **60** | **70** | **20** | **40** | **60** | **70** | **20** | **40** | **60** | **70** | **20** | **40** | **60** | **70** |
| **MDD  (F32-33)** | **Very high risk** | 24 | 68 | 67 | 73 | 29 | 36 | 31 | 26 | 17 | 43 | 45 | 44 | 20 | 44 | 58 | 15 | 78 | 91 | 89 | 89 | 29 | 37 | 50 | 67 |
|  | **High risk** | 0 | 21 | 99 | 97 | 15 | 58 | 133 | 48 | 0 | 26 | 88 | 71 | 8 | 51 | 37 | 8 | 0 | 68 | 98 | 95 | 9 | 65 | 49 | 33 |
|  | **Moderate risk** | 0 | 0 | 7 | 24 | 5 | 28 | 58 | 55 | - | 0 | 7 | 5 | - | 20 | 17 | 17 | 0 | 0 | 20 | 28 | 6 | 27 | 29 | 24 |
|  | **Low risk** | - | 0 | 0 | 0 | - | 7 | 33 | 40 | - | - | 0 | 0 | - | - | 8 | 7 | - | 0 | 0 | 0 | - | 15 | 19 | 17 |
|  | **Very low risk** | 0 | 0 | 0 | 0 | 2 | 7 | 19 | 25 | 0 | 0 | 0 | 0 | 2 | 6 | 4 | 5 | 0 | 0 | 0 | 0 | 4 | 10 | 13 | 13 |
| **Schizophrenia (F20)** | **Very high risk** | 7 | 36 | 40 | 33 | 7 | 8 | 3 | 3 | 8 | 72 | 100 | 97 | 0 | 13 | - | 0 | 3 | 11 | 14 | 15 | 2 | 2 | 1 | 3 |
|  | **High risk** | 0 | 0 | 0 | 0 | 3 | 5 | 1 | 2 | 0 | 0 | 12 | 9 | 0 | 10 | 8 | 5 | 0 | 0 | 0 | 0 | 0 | 1 | 1 | 1 |
|  | **Moderate risk** | 0 | 0 | 0 | 0 | 1 | 2 | 1 | 1 | - | 0 | 0 | 0 | - | 2 | 3 | 3 | 0 | 0 | 0 | 0 | 0 | 0 | 0 | 0 |
|  | **Low risk** | - | 0 | 0 | 0 | - | 1 | 1 | 0 | - | - | 0 | 0 | - | - | 1 | 1 | - | 0 | 0 | 0 | - | 0 | 0 | 0 |
|  | **Very low risk** | 0 | 0 | 0 | 0 | 1 | 1 | 1 | 1 | 0 | 0 | 0 | 0 | 1 | 2 | 1 | 1 | 0 | 0 | 0 | 0 | 0 | 0 | 0 | 0 |
| **Bipolar disorder (F31)** | **Very high risk** | 2 | 5 | 8 | 7 | 5 | 19 | 13 | 8 | 2 | 11 | 18 | 16 | 3 | 12 | 31 | 20 | 0 | 2 | 5 | 7 | 1 | 5 | 9 | 17 |
|  | **High risk** | 1 | 1 | 3 | 2 | 2 | 5 | 7 | 2 | 0 | 3 | 5 | 2 | 0 | 6 | 11 | 4 | 0 | 0 | 1 | 1 | 0 | 2 | 3 | 3 |
|  | **Moderate risk** | 0 | 1 | 2 | 1 | 1 | 2 | 2 | 2 | - | 0 | 1 | 0 | - | 2 | 1 | 3 | 0 | 0 | 0 | 1 | 0 | 0 | 1 | 2 |
|  | **Low risk** | - | 0 | 0 | 0 | - | 1 | 1 | 1 | - | - | 0 | 0 | - | - | 1 | 0 | - | 0 | 0 | 0 | - | 0 | 0 | 0 |
|  | **Very low risk** | 0 | 0 | 0 | 0 | 0 | 1 | 1 | 1 | 0 | 0 | 0 | 0 | 0 | 1 | 0 | 0 | 0 | 0 | 0 | 0 | 0 | 0 | 0 | 0 |
| **Anxiety related disorders (F40-41)** | **Very high risk** | 36 | 44 | 35 | 32 | 141 | 137 | 98 | 96 | 14 | 21 | 16 | 11 | 30 | 14 | 23 | 9 | 1 | 16 | 38 | 33 | 5 | 45 | 47 | 54 |
|  | **High risk** | 18 | 54 | 36 | 35 | 78 | 130 | 99 | 79 | 0 | 15 | 15 | 13 | 8 | 31 | 19 | 10 | 1 | 5 | 17 | 28 | 1 | 21 | 49 | 49 |
|  | **Moderate risk** | 0 | 15 | 47 | 31 | 47 | 80 | 77 | 67 | - | 0 | 5 | 3 | - | 5 | 6 | 1 | 0 | 2 | 11 | 10 | 1 | 9 | 21 | 27 |
|  | **Low risk** | - | 0 | 1 | 13 | - | 29 | 62 | 53 | - | - | 0 | 1 | - | - | 4 | 3 | - | 0 | 0 | 4 | - | 4 | 12 | 16 |
|  | **Very low risk** | 0 | 0 | 0 | 0 | 10 | 21 | 29 | 38 | 0 | 0 | 0 | 0 | 2 | 2 | 2 | 2 | 0 | 0 | 0 | 0 | 1 | 3 | 7 | 13 |
| **Stress related disorders  (F43)** | **Very high risk** | 34 | 41 | 34 | 36 | 29 | 48 | 30 | 20 | 10 | 16 | 9 | 3 | 22 | 26 | 7 | 0 | 2 | 9 | 31 | 18 | 1 | 29 | 11 | 2 |
|  | **High risk** | 7 | 25 | 51 | 67 | 20 | 48 | 46 | 30 | 0 | 11 | 14 | 10 | 4 | 19 | 14 | 7 | 0 | 9 | 7 | 12 | 1 | 13 | 15 | 5 |
|  | **Moderate risk** | 0 | 3 | 17 | 17 | 9 | 27 | 30 | 26 | - | 0 | 3 | 3 | - | 5 | 4 | 3 | 0 | 0 | 9 | 8 | 0 | 9 | 9 | 2 |
|  | **Low risk** | - | 0 | 0 | 2 | - | 10 | 24 | 19 | - | - | 0 | 0 | - | - | 1 | 2 | - | 0 | 0 | 1 | - | 4 | 6 | 2 |
|  | **Very low risk** | 0 | 0 | 0 | 0 | 2 | 8 | 13 | 14 | 0 | 0 | 0 | 0 | 2 | 2 | 1 | 2 | 0 | 0 | 0 | 0 | 0 | 2 | 3 | 1 |
| **Mental disorders related to alcohol abuse  (F10)** | **Very high risk** | 7 | 12 | 22 | 13 | 26 | 38 | 20 | 15 | 8 | 21 | 23 | 16 | 4 | 14 | 33 | 0 | 4 | 6 | 12 | 14 | 1 | 16 | 14 | 11 |
|  | **High risk** | 3 | 15 | 5 | 7 | 12 | 18 | 18 | 11 | 0 | 27 | 23 | 17 | 2 | 30 | 12 | 5 | 0 | 4 | 4 | 6 | 0 | 5 | 11 | 11 |
|  | **Moderate risk** | 0 | 0 | 10 | 8 | 5 | 12 | 12 | 10 | - | 0 | 16 | 17 | - | 10 | 9 | 6 | 0 | 0 | 3 | 6 | 0 | 3 | 9 | 8 |
|  | **Low risk** | - | 0 | 0 | 1 | - | 2 | 11 | 7 | - | - | 0 | 0 | - | - | 5 | 5 | - | 0 | 0 | 1 | - | 1 | 5 | 7 |
|  | **Very low risk** | 0 | 0 | 0 | 0 | 1 | 3 | 4 | 4 | 0 | 0 | 0 | 0 | 1 | 4 | 6 | 3 | 0 | 0 | 0 | 0 | 0 | 1 | 4 | 6 |
| **Irritable bowel syndrome  (K58)** | **Very high risk** | 1 | 2 | 4 | 4 | 6 | 7 | 6 | 4 | 0 | 1 | 3 | 2 | 3 | 0 | 0 | 0 | 2 | 11 | 16 | 14 | 12 | 34 | 13 | 11 |
|  | **High risk** | 0 | 2 | 4 | 6 | 3 | 5 | 8 | 7 | 1 | 1 | 3 | 5 | 0 | 8 | 5 | 7 | 2 | 7 | 16 | 14 | 5 | 23 | 22 | 13 |
|  | **Moderate risk** | 2 | 2 | 2 | 3 | 2 | 4 | 6 | 6 | - | 2 | 1 | 3 | - | 3 | 5 | 3 | 1 | 7 | 8 | 9 | 3 | 16 | 13 | 9 |
|  | **Low risk** | - | 1 | 2 | 2 | - | 3 | 4 | 6 | - | - | 2 | 2 | - | - | 5 | 6 | - | 6 | 8 | 7 | - | 10 | 13 | 8 |
|  | **Very low risk** | 0 | 0 | 0 | 1 | 1 | 1 | 4 | 4 | 0 | 0 | 0 | 0 | 1 | 1 | 2 | 2 | 0 | 0 | 2 | 4 | 2 | 6 | 8 | 6 |
| **Overweight and obesity (E66)** | **Very high risk** | 10 | 16 | 26 | 34 | 21 | 70 | 91 | 89 | 2 | 3 | 6 | 4 | 3 | 22 | 7 | 26 | 0 | 3 | 12 | 16 | 0 | 22 | 41 | 35 |
|  | **High risk** | 5 | 10 | 22 | 30 | 12 | 44 | 80 | 84 | 3 | 2 | 7 | 5 | 2 | 10 | 21 | 7 | 0 | 1 | 9 | 13 | 0 | 8 | 43 | 41 |
|  | **Moderate risk** | 14 | 9 | 18 | 31 | 8 | 41 | 73 | 83 | - | 3 | 4 | 5 | - | 6 | 8 | 11 | 0 | 2 | 7 | 17 | 0 | 9 | 31 | 37 |
|  | **Low risk** | - | 3 | 19 | 20 | - | 15 | 73 | 82 | - | - | 3 | 3 | - | - | 8 | 6 | - | 1 | 5 | 8 | - | 4 | 26 | 36 |
|  | **Very low risk** | 0 | 0 | 2 | 2 | 2 | 10 | 36 | 48 | 0 | 0 | 0 | 0 | 0 | 1 | 3 | 2 | 0 | 0 | 1 | 1 | 0 | 3 | 14 | 25 |
| **Gastroesophageal**  **reflux  (K21)** | **Very high risk** | 1 | 2 | 5 | 6 | 7 | 15 | 23 | 26 | 1 | 3 | 5 | 6 | 7 | 11 | 7 | 36 | 1 | 8 | 20 | 25 | 2 | 37 | 39 | 60 |
|  | **High risk** | 1 | 2 | 4 | 5 | 4 | 14 | 29 | 31 | 0 | 4 | 8 | 9 | 0 | 14 | 31 | 21 | 1 | 2 | 17 | 24 | 2 | 22 | 65 | 61 |
|  | **Moderate risk** | 0 | 1 | 3 | 5 | 4 | 9 | 25 | 27 | - | 2 | 6 | 8 | - | 11 | 17 | 15 | 0 | 5 | 12 | 20 | 1 | 18 | 42 | 47 |
|  | **Low risk** | - | 0 | 3 | 3 | - | 4 | 19 | 23 | - | - | 4 | 8 | - | - | 20 | 17 | - | 0 | 9 | 17 | - | 9 | 47 | 47 |
|  | **Very low risk** | 0 | 0 | 0 | 1 | 0 | 2 | 8 | 14 | 0 | 0 | 1 | 1 | 0 | 3 | 7 | 9 | 0 | 0 | 4 | 10 | 1 | 6 | 33 | 41 |
